# Independent judgment before viewing generative AI recommendations reduces susceptibility to misleading advice: a randomized scenario-based experiment with preservice teachers

**DOI:** 10.3389/fpsyg.2026.1936522

**Published:** 2026-09-28

**Authors:** Dong Yan

**Affiliations:** Department of Education, Graduate School, Sehan University, Yeongam-gun, Republic of Korea

**Keywords:** advice timing, AI-assisted decision-making, appropriate reliance, automation bias, generative AI recommendations, judgment–recommendation sequence, pedagogical judgment, preservice teachers

## Abstract

**Introduction:**

Generative AI can support preservice teachers’ pedagogical decisions, but fluent recommendations may also redirect attention away from diagnostic classroom evidence. We compared an independent-judgment-before-recommendation sequence with a recommendation-before-judgment sequence under expert-keyed and misleading generative AI recommendations.

**Methods:**

A three-arm randomized scenario-based experiment enrolled 240 preservice teachers from one Chinese university. Group A recorded an independent judgment before viewing an AI recommendation, Group B viewed the recommendation before its first recorded judgment, and Group C completed the tasks without AI as a contextual benchmark and did not enter the primary sequence × recommendation-quality hypothesis test. Groups A and B completed 12 fixed-order items, including six expert-keyed and six misleading recommendations. Expert-keyed joint accuracy—agreement with the expert-designated plan and rationale—was the primary outcome. After data-quality exclusions, 218 participants remained in the main analysis. Participant-clustered logistic generalized estimating equations tested sequence, recommendation quality, and their interaction.

**Results:**

Under misleading recommendations, Group A had higher expert-keyed joint accuracy than Group B, OR = 1.912, 95% CI [1.413, 2.589], *p* < 0.001. The estimate remained significant among all 160 randomized A/B participants with available outcomes, OR = 1.814, 95% CI [1.345, 2.448], *p* < 0.001, and was similar after version adjustment. The difference appeared in both final plan accuracy and final rationale accuracy. Group A was also less likely to reproduce the misleading recommendation completely, OR = 0.403, 95% CI [0.296, 0.549], *p* < 0.001. Under expert-keyed recommendations, the between-group contrast was not statistically significant, OR = 0.821, 95% CI [0.598, 1.128], *p* = 0.225.

**Discussion:**

Recording an independent judgment before viewing AI was associated with lower susceptibility to misleading recommendations in the selected controlled scenarios. The result supports further evaluation of independent-first workflows in teacher education, but it does not isolate a single cognitive mechanism or establish transfer to authentic classroom decision-making. These findings extend research on cognitive-forcing designs to contextualized pedagogical judgments that require both instructional action selection and evidence-based justification.

## 1 Introduction

Generative AI is moving into teacher education, lesson preparation, and pedagogical decision-making. Teacher-facing systems can assist with instructional planning, resource development, assessment, and feedback, but they also raise questions about accuracy, pedagogical fit, and the teacher’s responsibility for final decisions (Holmes and Tuomi, 2022; Celik et al., 2022; Kasneci et al., 2023; Miao and Holmes, 2023). These concerns are especially relevant when fluent natural-language recommendations conceal weak reasoning or a poor fit with classroom evidence.

Preservice teachers are a particularly relevant population because their pedagogical judgment is still developing. Such judgment requires them to notice classroom information, interpret student thinking and instructional conditions, and connect that evidence to an appropriate response (Seidel and Stürmer, 2014; König et al., 2022; Martin et al., 2022). A confident AI explanation may therefore shape how they frame a classroom problem before they have stabilized their own interpretation.

Research on generative AI in teacher education has focused mainly on lesson planning, resource generation, attitudes, and perceived usefulness. Preservice teachers report benefits alongside concerns about accuracy, curricular alignment, and overdependence (Lee and Zhai, 2024; Kalenda et al., 2025; Yadav et al., 2026). Process studies show that interaction traces can distinguish reflective iteration, linear extraction, ownership, and agency during GenAI-supported lesson design (Tran et al., 2026; Krushinskaia et al., 2026). What remains unclear is whether the judgment–recommendation sequence changes performance when recommendation quality is experimentally varied.

Reliance should vary with recommendation quality. Agreement with flawed advice can produce automation bias, whereas ignoring accurate advice can also impair performance (Parasuraman and Riley, 1997; Schemmer et al., 2023). The judgment–recommendation sequence may determine whether preservice teachers form an interpretation of classroom evidence before encountering AI input. Judging first may protect against misleading recommendations but also reduce revision when advice is correct (Buçinca et al., 2021; Tversky and Kahneman, 1974; Nickerson, 1998).

Previous cognitive-forcing studies have rarely examined pedagogical decisions that require both an instructional action and an evidence-based rationale. We compared two judgment–recommendation sequences while varying recommendation quality within participants: two AI groups received six expert-keyed and six misleading fixed recommendations, and a no-AI group provided a contextual benchmark. The primary question was whether recording an independent judgment before AI would reduce susceptibility to misleading recommendations without a statistically detectable disadvantage under expert-keyed recommendations.

## 2 Theoretical and empirical background

### 2.1 Automation bias and reliance on AI advice

Automation bias occurs when a fluent recommendation discourages users from checking other evidence. The present study focuses on misuse: agreement with a credible-seeming recommendation that conflicts with the pedagogical scenario (Parasuraman and Riley, 1997).

In pedagogical judgment, early advice may influence not only the answer but the initial problem representation by directing attention toward particular student cues, classroom conditions, or instructional goals. The risk is greatest when a recommendation is professionally phrased yet pedagogically misleading.

Responses to algorithmic advice vary across users and contexts, ranging from aversion after observed errors to appreciation of algorithmic recommendations (Dietvorst et al., 2015; Logg et al., 2019). This variability makes recommendation quality—not reliance in the abstract—the relevant basis for evaluation.

### 2.2 Behavioral alignment as an indicator of quality-contingent reliance

Appropriate reliance provides the conceptual framework, whereas the study measures final-response alignment. Alignment can be useful when a recommendation is correct and harmful when it is wrong; non-alignment with misleading advice can reflect either an expert-keyed response or a different error.

Schemmer et al. (2023) described appropriate reliance as following correct advice and relying on one’s own judgment when advice is incorrect. Here, matching scores show whether the selected plan and rationale aligned with the recommendation. Expert-keyed joint accuracy and the mutually exclusive response categories provide the stronger evidence about whether that alignment was beneficial or harmful.

In the recommendation-first group, agreement cannot establish advice uptake because the first recorded response followed AI exposure. The matching indicators are therefore interpreted as observable response patterns rather than measures of a private acceptance process.

### 2.3 Judgment–recommendation sequence: protective effects and anchoring risks

The randomized manipulation changed the judgment–recommendation sequence: whether an independent response preceded the AI recommendation. This operationalized advice timing by placing the recommendation before or after an initial pedagogical judgment. In the independent-first condition, participants formed a response before viewing AI advice, creating a concrete position against which the recommendation could be compared. This resembles a cognitive forcing step (Buçinca et al., 2021).

The proposed protective pathway is that an independent response requires participants to construct an initial representation of the pedagogical problem and a comparison standard before AI exposure. The later recommendation can then be evaluated against both the scenario evidence and the self-generated plan–rationale pair, potentially increasing detection of conflicts when advice is misleading. In pedagogical judgment, this comparison concerns not only the instructional action but also the evidence used to justify it. A competing pathway is that forming an initial response also creates commitment to that response; when later advice conflicts with the initial position, anchoring or confirmation bias may reduce revision even when the advice is expert-keyed. Attention allocation, evidence evaluation, self-generation, uncertainty awareness, and commitment to an initial response are therefore plausible mechanisms, but they were not measured directly and are not treated as demonstrated mediators. Figure 1 summarizes these hypothesized competing pathways.

**Figure 1:**
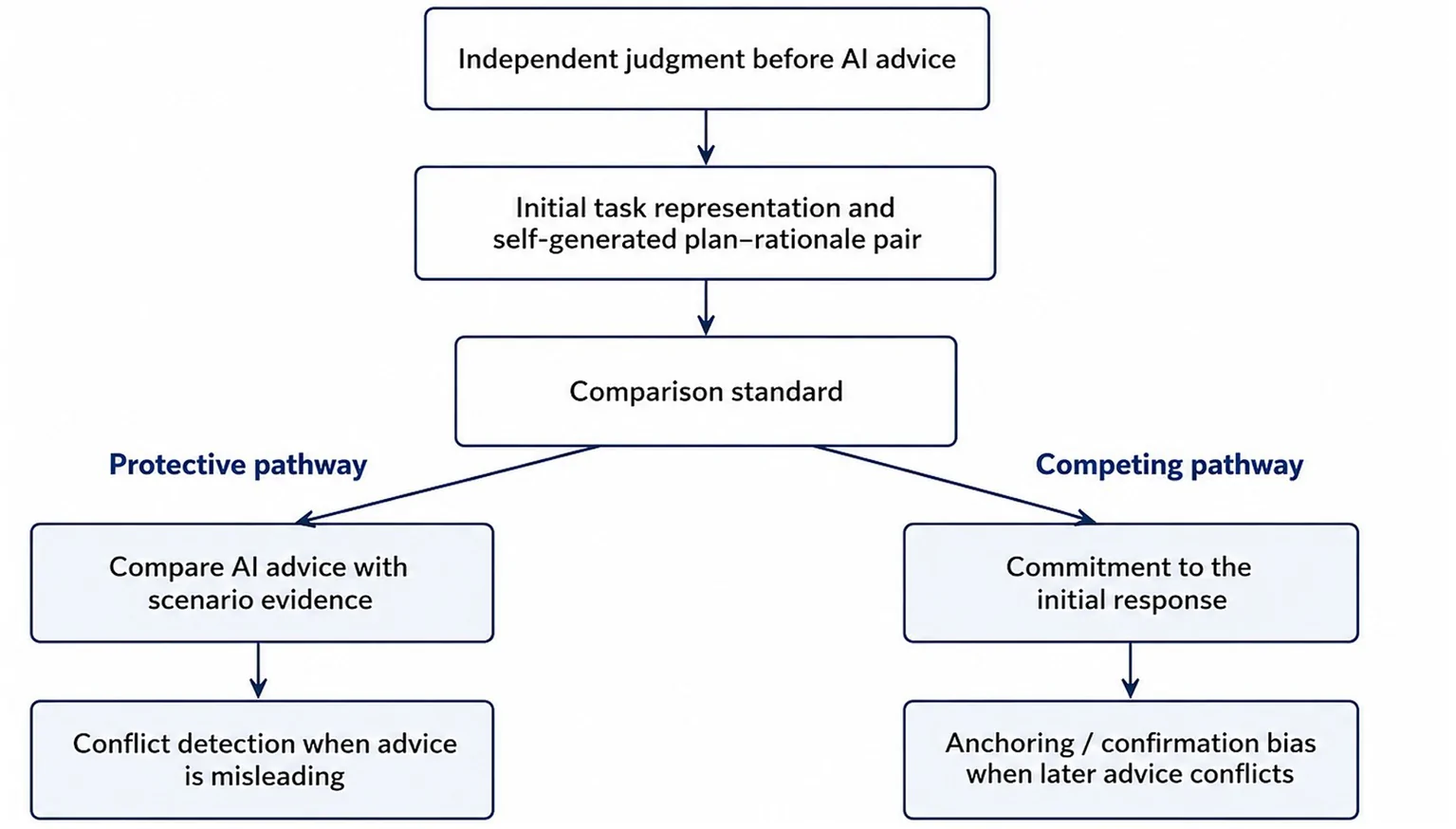
Hypothesized competing pathways following an independent pre-AI judgment. The figure presents theoretical pathways only. The study did not directly measure task representation, conflict detection, uncertainty awareness, anchoring, or confirmation bias.

The protective account predicts that judging first will improve final accuracy and reduce complete reproduction of misleading recommendations. A competing anchoring account predicts that an initial response may make revision less likely when later advice is expert-keyed (Tversky and Kahneman, 1974; Nickerson, 1998; Soll and Larrick, 2009).

Crossing the judgment–recommendation sequence with recommendation quality made it possible to examine both possibilities in the same experiment: protection under misleading advice and possible rigidity under expert-keyed advice.

### 2.4 Contextualized pedagogical judgment among preservice teachers

Pedagogical judgment requires more than recall of educational facts. Preservice teachers must notice classroom evidence, interpret what it means, and select an instructional response. Professional vision and teacher-noticing research treats these situation-specific processes as central to developing expertise (Seidel and Stürmer, 2014; König et al., 2022; Martin et al., 2022; Grub et al., 2020).

This task is demanding for preservice teachers because general pedagogical principles must be applied to ambiguous evidence and competing options. AI recommendations may support that translation or redirect attention toward salient but less diagnostic cues.

Unlike factual advice-taking tasks, pedagogical judgment requires coordination between an instructional action and the interpretation that justifies it. A plan can appear defensible while its rationale rests on a misreading of student evidence. The plan–rationale format therefore allowed the study to test whether the sequence affected both the selected instructional plan and the rationale used to justify it.

### 2.5 The present study, hypotheses, and research questions

The study compared an independent-first sequence with a recommendation-first sequence and used a no-AI group as a contextual benchmark. Because recommendation quality was undefined for the no-AI group, the core sequence × recommendation-quality model included only Groups A and B.

The hypotheses and research questions distinguished the primary accuracy outcome from complete reproduction of misleading advice and secondary confidence–accuracy correspondence.

*H1*: Under misleading AI recommendations, the independent-first sequence will show higher expert-keyed joint accuracy than the recommendation-first sequence.

*RQ1a*: Under expert-keyed AI recommendations, does the judgment–recommendation sequence affect expert-keyed joint accuracy?

*RQ1b*: Under expert-keyed AI recommendations, does the judgment–recommendation sequence affect behavioral alignment with the recommendation?

*H2a*: Under misleading AI recommendations, the recommendation-first sequence will show more complete matching with the misleading recommendation than the independent-first sequence.

*RQ2*: Under misleading AI recommendations, does the judgment–recommendation sequence affect confidence–accuracy correspondence?

The approximately two-week no-AI post-test was analyzed separately as a supplementary exploratory outcome.

## 3 Methods

### 3.1 Research design

The study used a three-arm randomized scenario-based experiment. After the eligible and consenting roster of 240 participants had been assembled, the researcher generated Excel random numbers and assigned 80 participants to each condition before the baseline phase.

The randomized manipulation compared two judgment–recommendation sequences. Group A made an independent pedagogical judgment before viewing the generative AI recommendation, whereas Group B viewed the recommendation before its first recorded judgment; Group C completed the same scenarios without AI. The term advice timing refers to this ordering, but the manipulation also differed in whether participants generated a pre-AI response, formed an initial commitment, and encountered a neutral review stage. The experiment therefore estimates the effect of the independent-first versus recommendation-first procedure as implemented, rather than an isolated temporal-distance effect.

All participants completed six no-AI baseline items. Groups A and B then completed 12 main items. Within each AI group, the first 40 assigned participant numbers received Version X and the next 40 received Version Y to maintain equal quotas. Each version contained six expert-keyed and six misleading recommendations, with quality reversed by item. All participants received the 12 main items in the same fixed order. Approximately 2 weeks later, participants were invited to complete six no-AI post-test items.

Recommendation quality was not defined for Group C. Group C therefore did not enter the core 2 × 2 timing × recommendation-quality model and was used only in planned, item-adjusted benchmark comparisons (Table 1, Figure 2).

**Table 1:** Experimental conditions and task structure.

Condition	Sequence	AI shown?	Main items	Recommendation quality	Analysis role
Group A: independent-first	Independent judgment → AI recommendation → final judgment	Yes	12	6 expert-keyed + 6 misleading	Core 2 × 2 GEE; planned benchmark comparisons
Group B: recommendation-first	AI recommendation → first judgment → neutral review → final judgment	Yes	12	6 expert-keyed + 6 misleading	Core 2 × 2 GEE; planned benchmark comparisons
Group C: no-AI benchmark	First judgment → neutral review → final judgment	No	12	Not applicable	Planned no-AI benchmark comparisons only

Group C received no AI recommendation and was not included in the sequence × recommendation-quality model.

Version X and Version Y each included 40 participants within Groups A and B at assignment. Both versions contained six expert-keyed and six misleading recommendation items; recommendation quality was reversed by item. The 12 main items were presented in a fixed order.

**Figure 2:**
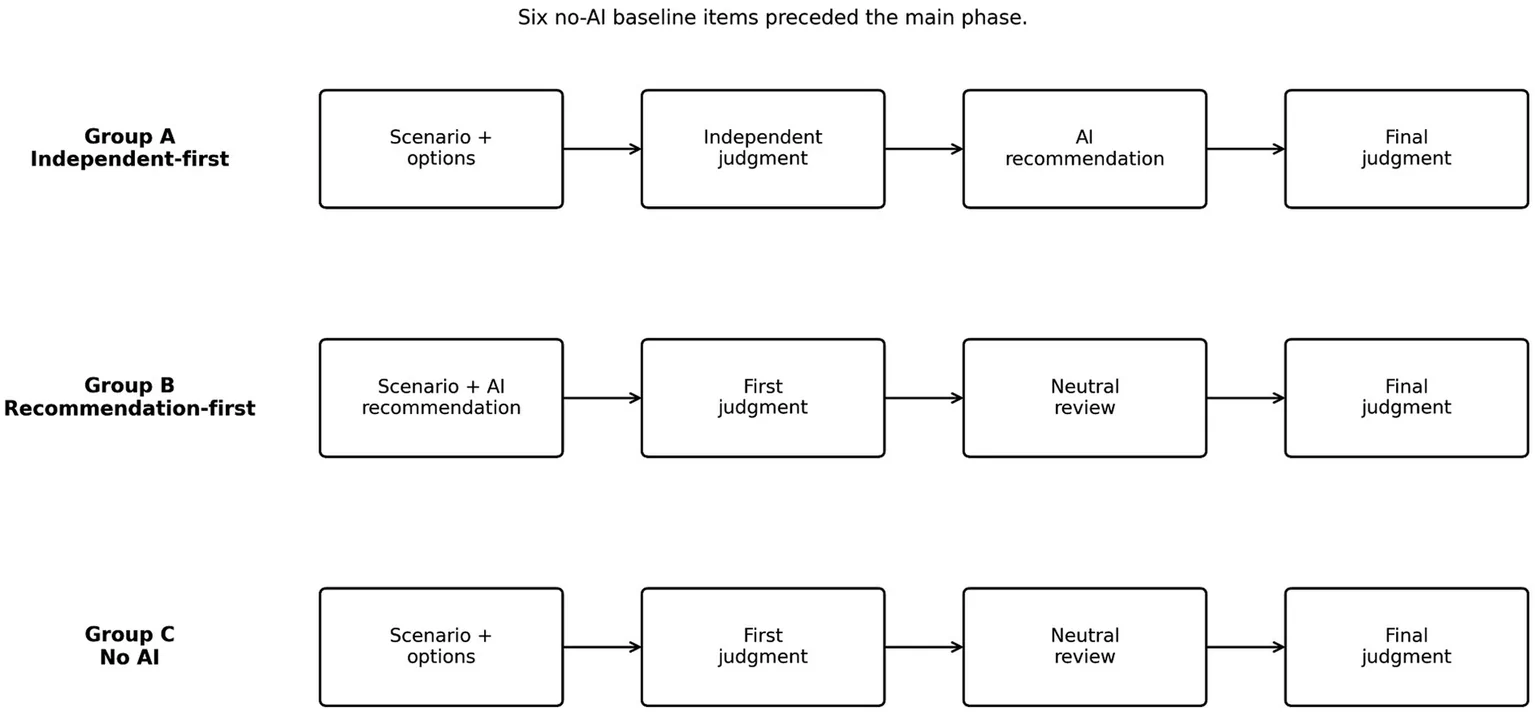
Experimental procedure and judgment–recommendation sequence. Group A completed scenario review and an independent plan, rationale, and confidence judgment before viewing the AI recommendation and then submitted a final judgment. Group B viewed the AI recommendation with the scenario before its first recorded plan, rationale, and confidence judgment, followed by a neutral review and final judgment. Group C received no AI recommendation and completed a first judgment, the same neutral review used in Group B, and a final judgment. The neutral review contained no new recommendation, answer key, or correctness feedback.

### 3.2 Participants and data collection

A total of 240 preservice teachers participated, with 80 assigned to each condition. They were recruited from teacher-education programs at one comprehensive university in China. The randomized cohort included 163 undergraduates (71 third-year and 92 fourth-year students) and 77 master’s students (47 first-year and 30 second-year students). Programs included education, teacher education, primary education, curriculum and instruction, educational technology, and subject teaching in mathematics, Chinese, and English. In this study, preservice teachers refers to students completing formal teacher-education coursework before entry into full-time teaching. Course instructors distributed recruitment information, but participation was voluntary.

Participants had to be at least 18 years old, enrolled in teacher education or an education-related program, able to read the Chinese materials, have prior exposure to basic pedagogical theory, and provide informed consent. Background variables included major, year of study, age, gender, teaching practicum experience, AI-use frequency, and prior use of AI for teaching-related tasks.

No formal *a priori* power analysis was conducted. The available sample was determined by the three-arm design, repeated item-level observations, and recruitment feasibility. In response to the observed uncertainty, the expert-keyed-advice comparison is interpreted through its effect estimate and confidence interval rather than as evidence of equivalence. The study can evaluate the direction and robustness of the focal misleading-advice contrast, but it was not designed as an equivalence or non-inferiority trial.

The experiment was administered online through Wenjuanxing. Before beginning the experiment, participants received information about the study purpose, task procedure, data use, anonymity, confidentiality, and their right to withdraw. Participation or withdrawal had no effect on course grades, course evaluation, or other academic rights. All participant data were de-identified before analysis.

### 3.3 Scenario-based judgment materials

The materials were developed from 40 candidate scenario-based items: 20 main items, 10 baseline items, and 10 post-test items. Seven experts independently rated each item on 13 content and recommendation-quality dimensions. Their expertise covered educational psychology and learning, teacher education, curriculum and instruction, classroom diagnosis, educational measurement, educational technology and AI in education, and school-based teaching research. The dimensions covered classroom authenticity, information sufficiency, answer-key clarity, construct relevance, expert-keyed-recommendation accuracy and contextual fit, misleading-recommendation error degree and surface plausibility, misleading potential, and language comparability. Experts also provided a separate overall recommendation concerning item retention or revision. Ratings of 4 or 5 on the five-point scale were treated as agreement for CVI calculations (Polit et al., 2007; Yusoff, 2019). Eight teacher-education students then completed cognitive interviews following a response-process approach (Wolcott et al., 2020), and a pilot study with 48 participants produced 1,920 item-level responses for inspection of difficulty, joint accuracy, response time, recommendation matching, and the credibility gap between expert-keyed and misleading recommendations. The final set contained 12 main items, six baseline items, and six post-test items. Item identifiers, item-level CVI summaries, answer keys, cognitive-interview summaries, pilot results, and fixed recommendation texts are reported in the Supplementary material.

Candidate answer keys were drafted from the item blueprint and the evidence chain specified for each scenario, then evaluated independently by the seven experts. The keyed plan and rationale represented the most defensible response to the evidence supplied in the scenario, rather than a context-free universal answer. Items were prioritized for revision when agreement on either keyed component fell below 80% or when experts identified an equally defensible alternative. Final selection considered content coverage, answer-key agreement, cognitive-interview evidence, item difficulty, response time, misleading-advice plausibility, and comparability between the expert-keyed and misleading advice texts. The expert key is therefore a panel-based content judgment, not an externally validated or uniquely correct classroom solution. Development-stage concerns and the final disposition of retained items are reported in the Supplementary material.

Across the 12 retained main items, mean expert ratings on the five-point scale were 4.27 for expert-keyed-advice accuracy, 4.31 for expert-keyed-advice contextual fit, 4.36 for the degree of error in the misleading advice, 4.31 for surface plausibility, 4.16 for language comparability, and 4.27 for misleading potential. The mean composite AI-quality CVI across the retained main items was 0.965. One retained item showed weaker pilot balance and was retained to preserve coverage of peer-feedback judgment; excluding that item did not materially change the focal timing estimate. The Supplementary material separates development-stage observations from the final materials used in the formal experiment. Across the full candidate pool, Kendall’s coefficient of concordance provided an additional ordinal agreement check beyond CVI. For the eight scenario, answer-key, and retainability dimensions rated across 40 candidate items, W ranged from 0.599 to 0.745; for the six AI-recommendation dimensions rated across the 20 main candidate items, W ranged from 0.624 to 0.738. All concordance tests were significant at *p* < 0.001. These values indicate moderate-to-strong agreement in the experts’ relative evaluations across the development pool; CVI remained the primary index of whether ratings met the prespecified acceptability threshold. Full coefficients are reported in Supplementary Section S3.1.

Each item contained a classroom scenario, four teaching plan options, and four pedagogical rationale options. Participants were required to select one teaching plan and one rationale. Each item had a predetermined expert answer key. The use of scenario-based materials was intended to assess contextualized pedagogical judgment rather than abstract recall of educational theory.

The primary scoring unit was joint correctness. An item was coded as jointly correct only when both the selected teaching plan and the selected pedagogical rationale matched the expert answer key. If either the teaching plan or the rationale was incorrect, the joint accuracy score for that item was coded as 0.

For example, one retained scenario described a student who rarely volunteered during whole-class questioning but showed note-taking, task completion, and evidence of participation in other formats. The expert-keyed response treated silence alone as insufficient evidence of low engagement, whereas the misleading recommendation inferred low engagement from limited public speaking and emphasized random calling. This contrast illustrates how a superficially plausible recommendation could conflict with the fuller evidence in the scenario.

### 3.4 AI recommendation texts and version control

The formal experiment used fixed recommendations initially generated with GPT-4 and subsequently revised by the researcher rather than live model outputs. GPT-4 generated the initial drafts on December 20, 2025. The researcher revised them against the item blueprint, expert answer keys, and predefined error mechanisms so that participants assigned to the same version received identical recommendations. The exact original interface prompt and sampling parameters were not archived; Supplementary Section S2.3 reconstructs the documented generation instructions and describes the revision procedure.

Each main item had one expert-keyed recommendation and one recommendation containing a predefined pedagogical error. The expert-keyed recommendation matched the panel-designated teaching plan and rationale. The misleading recommendation contained one central pedagogical error but retained a professional, coherent style. The expert panel reviewed accuracy, contextual fit, degree of error, surface plausibility, misleading potential, and language comparability. Texts were revised when the intended error was not clearly codable, when experts judged surface plausibility or misleading potential to be too low, or when paired texts differed noticeably in length, tone, or professionalism. Because the same panel reviewed the answer keys and the recommendations, this evidence reflects content agreement within the study framework rather than independent external validation; no second panel separately validated the finalized recommendation-quality classifications.

Version X and Version Y reversed advice quality at the item level. The archived assignment file records Version X for the first 40 participant IDs and Version Y for the next 40 within each AI group. At assignment, Group A contained 40 X-version and 40 Y-version participants, and Group B contained the same 40/40 allocation. After participant-level cleaning, the counts were 35 X and 37 Y in Group A and 38 X and 38 Y in Group B. Every participant in an AI condition encountered six expert-keyed and six misleading advice texts. The item sequence was fixed rather than randomized; item fixed-effect and version-adjusted sensitivity models were therefore used in addition to the primary model.

The recommendations were explicitly labeled as AI-generated. During the task, participants were not informed which recommendations were intended to be expert-keyed or misleading, and no item-level correctness feedback was provided. The formal experiment did not include an item-level rating of perceived AI authorship or recommendation credibility; evidence concerning plausibility and comparability came from expert review and pilot testing. After the experiment, participants were told that the recommendations were pre-generated, manually revised, and expert-reviewed rather than live outputs.

### 3.5 Experimental procedure

All participants first completed six no-AI baseline items. For each baseline item, participants read a classroom scenario, selected a teaching plan, selected a pedagogical rationale, and reported their confidence. No AI advice was shown during the baseline phase.

In Group A, participants read the scenario and response options and recorded an initial teaching plan, rationale, and confidence rating. The AI recommendation was then displayed once at the designated stage, followed by the final-response fields for plan, rationale, and confidence. The initial response was therefore a genuine pre-AI judgment.

In Group B, participants saw the scenario and AI recommendation before their first recorded plan, rationale, and confidence rating. They then received the neutral prompt, “Please reread the scenario and options. You may retain or revise your original judgment,” before submitting the final response. The recommendation was not restated in the neutral prompt or final-response fields, and no answer key or correctness feedback was provided.

Group C never saw an AI recommendation. Participants made a first judgment, received the same neutral review prompt used in Group B, and then submitted a final judgment. The prompt provided no explanation, feedback, or correct answer. Because Group A proceeded from the displayed recommendation directly to the final-response fields whereas Group C received an explicit neutral review prompt, Group A–Group C comparisons are treated as contextual benchmarks rather than a pure causal contrast between AI exposure and no AI.

Approximately 2 weeks later, all randomized participants were invited again to complete six no-AI post-test items; completion was voluntary rather than required. These items used new classroom scenarios and required participants to independently select a teaching plan, select a pedagogical rationale, and report confidence. No AI advice was provided in the post-test phase.

### 3.6 Measures

The primary outcome was expert-keyed joint accuracy. An item was coded 1 only when both the final teaching plan and final pedagogical rationale matched the expert answer key; all other combinations were coded 0.

Final plan accuracy and final rationale accuracy were also coded separately as secondary decomposition outcomes. Each component was coded 1 when the final selection matched the corresponding expert key and 0 otherwise. These outcomes were used to determine whether the joint-accuracy pattern was present in the instructional action, the supporting rationale, or both.

A final-response matching score was derived from participants’ choices: 0 when neither response component matched the recommendation, 1 when either the teaching plan or rationale matched, and 2 when both matched. Dividing the score by 2 produced a 0–1 alignment score. In Group B, this measure describes response agreement because the first recorded judgment followed AI exposure.

The incorrect-advice non-alignment score was calculated as 1 minus the normalized matching score. Complete matching indicated that both final components reproduced the recommendation. Under expert-keyed recommendations, complete matching equals expert-keyed joint accuracy; under misleading recommendations, the mutually exclusive response categories were an expert-keyed joint response, complete reproduction of the misleading recommendation, or another/partially aligned incorrect response.

Harmful and beneficial answer changes were calculated only for Group A. Harmful change was an initially correct joint judgment that became incorrect after misleading advice; beneficial change was an initially incorrect joint judgment that became correct after expert-keyed advice. The denominator was all valid Group A item observations in the relevant advice-quality condition. These transitions cannot be identified in Group B because its first recorded response followed AI exposure.

Correct self-reliance under misleading advice was defined as a correct final joint judgment without complete matching to the misleading recommendation. Because complete matching with a misleading recommendation cannot also be jointly correct, this indicator is numerically equivalent to expert-keyed joint accuracy in the misleading-advice condition and is treated as descriptive.

Confidence was reported on a 0–100 scale, with 0 indicating no confidence and 100 indicating complete confidence. Absolute confidence–accuracy error was calculated by converting confidence to a 0–1 scale and taking the absolute difference between confidence and final joint correctness: |confidence / 100 − final joint correctness|. Signed confidence bias was the mean of confidence / 100 − correctness, with positive values indicating overconfidence. The Brier score was the mean squared difference between confidence / 100 and binary correctness. These descriptive calibration indices complement the absolute-error analysis but do not constitute a latent measure of metacognitive monitoring (Schraw, 2009).

Response time was recorded for the available task phases. Participant-level main-phase timing is summarized descriptively in the Supplementary material; it was not included as a covariate because it occurred after assignment to the experimental sequence. The no-AI post-test joint accuracy score was used as an exploratory indicator of later unaided pedagogical judgment performance.

### 3.7 Data preprocessing and missing data

Cleaning rules were finalized during data preparation and applied before the focal outcome models were estimated; the analysis plan was not publicly preregistered. Checks covered experiment completion, an instructional attention check, extreme speeding, duplicate submissions, and item-level missingness.

Extreme speeding was operationalized as a total main-phase response time of less than 8 min. All 10 participants below this threshold were excluded, and no participant with a total time of 8 min or longer was excluded for speeding. The threshold and exclusion decisions were applied before the focal outcome models were estimated. To evaluate sensitivity to this preprocessing decision, the focal model was also estimated for all 160 randomized A/B participants with available primary-outcome data. The other documented participant-level exclusion reason was failure of the instructional attention check.

Of the 240 randomized participants, 22 were excluded from the main analysis sample: 12 for attention-check failure and 10 for extremely short response patterns. The cleaned main analysis sample therefore included 218 participants. By group, the cleaned main analysis sample included 72 participants in Group A, 76 in Group B, and 70 in Group C. Thirty participants did not complete the approximately two-week no-AI post-test, corresponding to an original post-test attrition rate of 12.5%. The no-AI post-test analysis included 192 participants who both passed the main analysis cleaning criteria and completed the post-test: 68 in Group A, 64 in Group B, and 60 in Group C.

Item-level missing data were handled using available observations for the relevant item-level indicators. A participant was not automatically removed from all analyses because of isolated item-level missingness, provided that the relevant outcome data were available. No additional participant-level exclusions were made for duplicate submissions, failure to complete the main experiment, or large-scale item-level missingness in the formal cleaning record.

For the post-test analysis, only participants who completed the approximately two-week no-AI post-test were included in the main post-test analysis. Sensitivity analyses were conducted using pessimistic and optimistic assumptions to evaluate how attrition affected interpretation of the exploratory post-test findings.

### 3.8 Statistical analysis

Primary analyses were conducted in IBM SPSS Statistics 27.0. During manuscript revision, the reported GEE coefficients, additional simple contrasts, participant-clustered benchmark models, and sensitivity analyses were reproduced in a separate software environment using Python 3.13 and statsmodels 0.14.6. De-identified item-level data and analysis syntax may be made available by the corresponding author in accordance with the approved data-sharing conditions.

Baseline group comparisons used ANOVA or chi-square tests as appropriate. Repeated item responses were analyzed with generalized estimating equations because observations were clustered within participants (Liang and Zeger, 1986; Zeger et al., 1988). GEE was selected because the primary estimand was the population-averaged sequence effect across participants for the 12 purpose-selected scenarios. A mixed-effects logistic model would estimate conditional participant- and item-specific effects and would be especially useful for generalizing to a random population of items; the present study instead treated the selected scenarios as a fixed task set and used categorical item fixed effects as a sensitivity analysis.

The primary estimand was the population-averaged difference between the independent-first and recommendation-first sequences for the 12 fixed experimental scenarios. Expert-keyed joint accuracy was analyzed with logistic GEE using a binomial distribution, logit link, exchangeable working correlation, participant ID as the clustering unit, and robust standard errors. Predictors were judgment–recommendation sequence, recommendation quality, their interaction, baseline joint accuracy, and item order. Group B under misleading advice was the reference condition. The Group A versus Group B contrast under expert-keyed advice was calculated from the Group A main effect and the interaction term.

The core 2 × 2 model included only Groups A and B. Group C had no advice-quality factor and was analyzed only in planned benchmark models.

The benchmark analysis used a five-level condition factor (Group C no AI, Group A misleading advice, Group B misleading advice, Group A expert-keyed advice, and Group B expert-keyed advice), baseline joint accuracy, and categorical item fixed effects in a participant-clustered logistic GEE. These contrasts contextualize performance relative to no AI and are separate from the 2 × 2 advice-timing model. Holm adjustment across the four benchmark contrasts did not change which comparisons were statistically significant.

Secondary plan and rationale accuracy outcomes were analyzed with the same participant-clustered logistic GEE specification. Matching and confidence–accuracy outcomes were modeled separately by recommendation quality. Gaussian GEE estimated marginal mean differences for the normalized matching score and absolute confidence–accuracy error, with robust standard errors; binary complete-matching outcomes were analyzed with logistic GEE. H1 was the primary hypothesis test and H2a was a directional secondary hypothesis. RQ1a, RQ1b, RQ2, benchmark comparisons, component decompositions, and post-test analyses were secondary or exploratory.

All participants received the same fixed item order, so item identity and serial position were confounded. The original model included linear item order. Because version allocation was quota-based rather than independently randomized, the focal sequence contrast was also estimated with version included and was reported alongside the original estimate. A categorical item-fixed-effect model was used as the principal item-adjusted analysis. Additional sensitivity analyses added prior teaching-related AI use and general AI-use frequency, used an independent working correlation, retained all randomized A/B participants with available outcomes, estimated the focal contrast separately within Versions X and Y, and repeated the model after excluding each item in turn.

The correct-advice simple contrast, item-adjusted benchmark model, all-randomized analysis, version-stratified estimates, and leave-one-item-out analyses were used to assess the stability of the focal sequence effect.

For Figure 3, percentile 95% confidence intervals around the observed condition means were calculated from 10,000 participant-level bootstrap resamples using a fixed random seed (20260710). Each resampled participant contributed all available item observations for the relevant condition.

**Figure 3:**
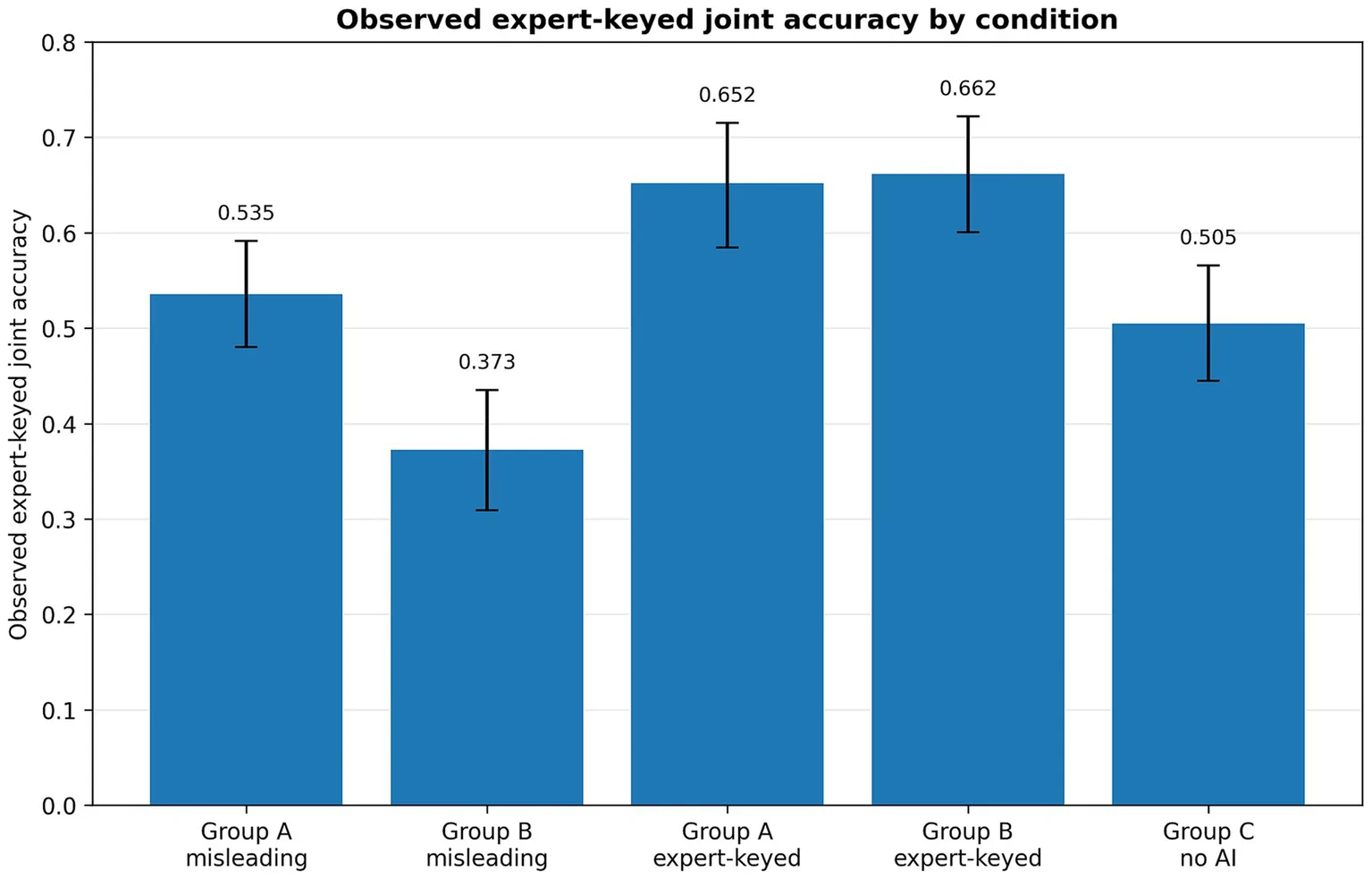
Observed expert-keyed joint accuracy with participant-clustered bootstrap 95% confidence intervals. Bars show observed item-level means; error bars are percentile 95% confidence intervals from 10,000 participant-level bootstrap resamples. Group C is shown as a contextual no-AI benchmark and was not included in the core sequence × recommendation-quality model. Inferential results are reported in Table 3 and Supplementary Section S5.3.

## 4 Results

### 4.1 Baseline equivalence and descriptive statistics

A total of 240 preservice teachers were randomized into the three experimental conditions, with 80 participants initially assigned to each group. During data preprocessing, 22 participants were excluded according to the cleaning rules established before the main statistical modeling: 12 failed the attention check and 10 showed extremely short response patterns. The cleaned main analysis sample therefore included 218 participants: 72 in Group A, 76 in Group B, and 70 in Group C. Thirty participants did not complete the approximately two-week no-AI post-test, corresponding to an original post-test attrition rate of 12.5%. The post-test analysis sample included 192 participants who passed the main cleaning criteria and completed the post-test: 68 in Group A, 64 in Group B, and 60 in Group C (Table 2).

**Table 2:** Sample flow and analysis samples.

Stage	Group A	Group B	Group C	Total
Randomized	80	80	80	240
Attention-check failure	4	2	6	12
Extremely short response pattern	4	2	4	10
Excluded in total	8	4	10	22
Included in main analysis	72	76	70	218
Original post-test not completed	6	13	11	30
Included in post-test analysis	68	64	60	192

Original post-test non-completion was calculated based on the 240 randomized participants. Post-test analysis included participants who passed main cleaning criteria and completed the no-AI post-test. Because some participants excluded from the main analysis also completed or did not complete the post-test, original post-test non-completion among randomized participants does not equal the difference between the cleaned main sample and the post-test analysis sample. At assignment, Version X/Y counts were 40/40 in both AI groups; after cleaning, the counts were 35/37 in Group A and 38/38 in Group B.

Descriptive baseline differences were generally small, although prior use of AI for teaching-related tasks differed across the cleaned groups, χ^2^ = 6.873, *p* = 0.032, and was therefore included in sensitivity analyses. For the cleaned A/B comparison, standardized mean differences were 0.032 for age, 0.149 for baseline joint accuracy, and 0.268 for prior teaching-related AI use. The corresponding omnibus tests for age, baseline joint accuracy, gender, AI-use frequency, and teaching-practicum experience were not statistically significant. Full background distributions are reported in Supplementary Section S5.1.

### 4.2 Core GEE logistic model for expert-keyed joint accuracy

The core 2 × 2 GEE included Groups A and B only. Expert-keyed joint accuracy was the binary outcome.

Observed expert-keyed joint accuracy was 0.535 in Group A and 0.373 in Group B under misleading advice. Under expert-keyed advice, the corresponding means were 0.652 and 0.662.

Under misleading advice, the independent-first sequence produced higher odds of expert-keyed joint accuracy than the recommendation-first sequence, log odds = 0.648, robust SE = 0.155, Wald χ^2^ = 17.61, OR = 1.912, 95% CI [1.413, 2.589], *p* < 0.001. The observed difference was 16.2 percentage points. Adjustment for version yielded a nearly identical estimate, OR = 1.901, 95% CI [1.403, 2.577], *p* < 0.001. The comparison also remained significant when all 160 randomized A/B participants with available item outcomes were retained, OR = 1.814, 95% CI [1.345, 2.448], *p* < 0.001, and in the categorical item-fixed-effect model, OR = 1.935, 95% CI [1.422, 2.633], *p* < 0.001 (Table 3)

**Table 3:** Core GEE logistic model predicting expert-keyed joint accuracy in Groups A and B.

Predictor/comparison	Log odds	Robust SE	Wald chi-square	OR	95% CI	p
Group A vs. Group B under misleading AI recommendation	0.648	0.155	17.61	1.912	[1.413, 2.589]	< 0.001
Expert-keyed AI recommendation vs. misleading AI recommendation within Group B	1.424	0.172	68.52	4.154	[2.965, 5.820]	< 0.001
Group A × expert-keyed AI interaction	−0.845	0.224	14.25	0.429	[0.277, 0.666]	< 0.001
Baseline joint accuracy (per 0.10 increase)	0.429	0.034	161.93	1.536	[1.438, 1.641]	< 0.001
Item order	0.010	0.015	0.45	1.010	[0.981, 1.040]	0.500
Group A vs. Group B under expert-keyed AI recommendation	−0.197	0.162	1.47	0.821	[0.598, 1.128]	0.225

The displayed coefficients are from the original participant-clustered GEE with a binomial distribution, logit link, exchangeable working correlation, and robust standard errors. Baseline joint accuracy and item order were covariates. Because version was quota-assigned, the focal contrast was also estimated with version included: OR = 1.901, 95% CI [1.403, 2.577], p < 0.001. The baseline coefficient is presented for a 0.10 increase on the 0–1 scale; the corresponding unscaled coefficient was 4.293.

Within Group B, expert-keyed joint accuracy was substantially higher under expert-keyed than misleading advice, OR = 4.154, 95% CI [2.965, 5.820], *p* < 0.001. The sequence × recommendation-quality interaction was significant, OR = 0.429, 95% CI [0.277, 0.666], *p* < 0.001. Under expert-keyed advice, the Group A versus Group B simple contrast was not statistically significant, OR = 0.821, 95% CI [0.598, 1.128], *p* = 0.225. Item order was not significant, OR = 1.010, 95% CI [0.981, 1.040], *p* = 0.500.

Decomposing the joint outcome showed that the misleading-recommendation difference was present in both components. In version-adjusted models, Group A had higher final plan accuracy than Group B, 0.608 versus 0.439, OR = 1.938, 95% CI [1.453, 2.586], *p* < 0.001, and higher final rationale accuracy, 0.585 versus 0.406, OR = 2.028, 95% CI [1.482, 2.775], *p* < 0.001. Under expert-keyed recommendations, neither the plan contrast, OR = 0.941, 95% CI [0.677, 1.308], *p* = 0.717, nor the rationale contrast, OR = 0.728, 95% CI [0.522, 1.016], *p* = 0.062, was statistically significant. Version-adjusted component contrasts are reported in Supplementary Section S5.3.

### 4.3 Behavioral alignment with AI recommendations

Behavioral alignment was examined through complete matching and the normalized matching score. The mutually exclusive response categories under misleading recommendations distinguish expert-keyed responses from complete reproduction of the misleading recommendation and other errors.

Under expert-keyed recommendations, the observed alignment score was 0.767 in Group A and 0.781 in Group B, adjusted difference = −0.038, SE = 0.022, *p* = 0.086. Complete matching was 0.652 and 0.662, respectively, and was identical to expert-keyed joint accuracy in this condition; the core-model contrast was OR = 0.821, 95% CI [0.598, 1.128], *p* = 0.225.

Under misleading recommendations, complete reproduction of the misleading recommendation was less frequent in Group A than Group B, 0.258 versus 0.468, OR = 0.403, 95% CI [0.296, 0.549], *p* < 0.001, supporting H2a. The complementary non-alignment score was 0.702 versus 0.510, adjusted difference = 0.172, SE = 0.033, *p* < 0.001. The mutually exclusive distribution was: expert-keyed joint response, 0.535 versus 0.373; complete reproduction of the misleading recommendation, 0.258 versus 0.468; and another or partially aligned incorrect response, 0.207 versus 0.160.

Only Group A had a genuine pre-AI judgment. Across all valid Group A observations in the relevant conditions, the harmful-change proportion was 0.103 and the beneficial-change proportion was 0.195. These values are descriptive transition rates, not between-group effects (Table 4).

**Table 4:** Behavioral alignment with AI recommendations by recommendation quality and group.

Recommendation type	Indicator	Group A	Group B	Statistical test	*p*
Expert-keyed AI	Expert-keyed-advice alignment score	0.767	0.781	Adjusted difference = −0.038; SE = 0.022; Wald χ^2^ = 2.96	0.086
Expert-keyed AI	Complete matching / expert-keyed joint accuracy	0.652	0.662	Core-model contrast: OR = 0.821; 95% CI [0.598, 1.128]	0.225
Expert-keyed AI	Under-reliance (derived complement)	0.348	0.338	Descriptive only	Not inferentially tested
Misleading AI	Misleading-advice non-alignment score	0.702	0.510	Adjusted difference = 0.172; SE = 0.033; Wald χ^2^ = 27.14	< 0.001
Misleading AI	Complete matching with misleading advice	0.258	0.468	OR = 0.403; 95% CI [0.296, 0.549]	< 0.001
Misleading AI	Harmful answer change (all valid misleading-advice observations)	0.103	Not applicable	Group A only	Not inferentially tested
Expert-keyed AI	Beneficial answer change (all valid expert-keyed-advice observations)	0.195	Not applicable	Group A only	Not inferentially tested
Misleading AI	Other/partially aligned misleading response	0.207	0.160	Descriptive decomposition	Not inferentially tested

Matching scores are derived from final-response agreement with the AI plan and rationale. They do not identify internal advice uptake in Group B. Non-alignment with misleading advice may include either an expert-keyed response or an alternative error. Complete matching with expert-keyed advice is identical to expert-keyed joint accuracy. Harmful and beneficial changes were calculated only for Group A, using all valid observations in the relevant recommendation-quality condition as denominators.

### 4.4 Comparisons with the no-AI baseline group

Planned item-adjusted benchmark analyses placed the recommendation-first misleading-recommendation condition below the no-AI group, OR = 0.577, 95% CI [0.436, 0.764], *p* < 0.001. The independent-first misleading-recommendation condition did not differ significantly from no AI, OR = 1.125, 95% CI [0.864, 1.466], *p* = 0.380. Both expert-keyed-recommendation conditions were above the no-AI benchmark. These comparisons contextualize the AI conditions, but they are not interpreted as pure AI-versus-no-AI causal effects because Group A and Group C differed in review structure. Full estimates are reported in Supplementary Section S5.5.

### 4.5 Secondary confidence–accuracy analysis

RQ2 examined confidence–accuracy correspondence using absolute confidence–accuracy error, where lower values indicate a smaller discrepancy between stated confidence and final correctness.

Under misleading recommendations, Group A had a smaller absolute error than Group B, 0.419 versus 0.493, adjusted difference = −0.064, SE = 0.015, *p* < 0.001. This difference co-occurred with the group difference in accuracy. Mean confidence was identical when responses were jointly correct (0.767 in both groups) and similar when responses were jointly incorrect (0.633 in Group A and 0.648 in Group B). Signed confidence bias, interpreted here as calibration-in-the-large, was 0.170, 95% CI [0.120, 0.218], in Group A and 0.320, 95% CI [0.265, 0.375], in Group B. Brier scores were 0.230, 95% CI [0.209, 0.251], and 0.299, 95% CI [0.271, 0.327], respectively. Both groups therefore showed overconfidence, with a larger aggregate bias in Group B. Because absolute error and the Brier score incorporate accuracy, these findings are interpreted as secondary confidence–accuracy correspondence rather than definitive evidence of superior metacognitive monitoring. Binned calibration results and Supplementary Figure S2 are reported in Supplementary Section S5.4.

Absolute confidence–accuracy error was calculated as |confidence/100 − final joint correctness|. Lower values indicate a smaller discrepancy between confidence and correctness.

### 4.6 Exploratory no-AI post-test performance and sensitivity analysis

The approximately two-week no-AI post-test remained inconclusive under alternative attrition assumptions. Among the 192 cleaned completers, mean joint accuracy was 0.472 in Group A, 0.408 in Group B, and 0.452 in Group C. The A–B participant-level mean difference was 0.063, 95% CI [−0.017, 0.144], Hedges g = 0.268, *p* = 0.123; the item-adjusted participant-clustered GEE contrast was OR = 1.251, 95% CI [0.915, 1.710], *p* = 0.160. Follow-up completion did not differ significantly by group in the cleaned sample, χ^2^(2) = 4.233, *p* = 0.120. Completers and non-completers did not differ significantly in age or baseline joint accuracy; prior AI use for teaching-related tasks was less common among non-completers, but the difference did not reach statistical significance, χ^2^(1) = 3.229, *p* = 0.072. Under a pessimistic assumption that all missing post-tests were incorrect, the descriptive ordering remained A > C > B; under an optimistic assumption that all missing post-tests were correct, the ordering changed. Full effects and completion diagnostics are reported in Supplementary Section S6.1.

### 4.7 Robustness checks

The focal estimate remained statistically significant across all reported specifications. Whole-sample sensitivity estimates ranged from OR = 1.814 to OR = 1.984, whereas the version-specific estimates were OR = 2.152 for Version X and OR = 1.704 for Version Y. Across the 12 formal main items, overall cleaned-sample expert-keyed joint accuracy ranged from 0.454 for M16 to 0.616 for M20; no item exceeded 0.90 or fell below 0.10. In the version-adjusted leave-one-item-out models, the focal OR was smallest when M14 was excluded, OR = 1.857, 95% CI [1.362, 2.532], and largest when M16 was excluded, OR = 1.984, 95% CI [1.453, 2.710]; all 12 models remained significant (Supplementary Sections S5.2, S6.2).

To probe possible non-linear sequence trends, an exploratory GEE added centered linear and quadratic item-order terms to the core model. The linear order term remained non-significant (B = 0.010, SE = 0.015, *p* = 0.511), whereas the quadratic term was small but statistically significant (B = 0.010, SE = 0.005, *p* = 0.044). Because item identity and serial position were perfectly confounded, this curvature cannot distinguish non-linear practice or fatigue effects from item-specific difficulty. A parallel Gaussian GEE for log response time showed no linear or quadratic order trend (*p* = 0.699 and *p* = 0.833, respectively). Detailed descriptive values are reported in Supplementary Section S6.3.

An exploratory training-stage moderation model found no evidence that the sequence × recommendation-quality interaction differed between undergraduate and master’s students (three-way interaction *p* = 0.618). Under misleading recommendations, the independent-first versus recommendation-first contrast was in the same direction for undergraduates, OR = 1.832, 95% CI [1.272, 2.637], *p* = 0.001, and master’s students, OR = 2.031, 95% CI [1.211, 3.408], *p* = 0.007. These subgroup estimates are exploratory and less precise than the full-sample estimate because of the smaller stratum-specific samples (Supplementary Section S6.3).

## 5 Discussion

### 5.1 Principal findings

The advantage of the independent-first sequence was concentrated in trials where the AI recommendation was wrong. Participants who judged independently first had higher expert-keyed joint accuracy and were less likely to reproduce the misleading recommendation. The same pattern appeared in both the final instructional plan and the supporting rationale. The focal difference remained evident in the all-randomized analysis and in the version- and item-adjusted models.

The no-AI benchmark adds descriptive context to this pattern: the recommendation-first misleading-recommendation condition was below the unaided group, whereas the two expert-keyed-recommendation conditions were above it. Because the independent-first and no-AI procedures did not share an identical review structure, these benchmark contrasts should not be interpreted as isolating the causal effect of AI exposure.

### 5.2 Independent judgment before AI mattered most under misleading recommendations

One possible explanation is that the initial judgment required participants to generate a task representation and a plan–rationale pair before the recommendation appeared. That representation may have provided a comparison standard, making discrepancies between the recommendation and scenario evidence easier to detect. Cognitive forcing, deeper initial encoding, self-generation, retrieval, attentional allocation, evidence evaluation, and commitment to an initial answer are all viable explanations (Buçinca et al., 2021). The experiment varied these procedural components together and therefore identifies an effect of the implemented judgment–recommendation sequence, not a single confirmed cognitive mechanism.

The behavioral pattern was specific: the independent-first sequence coincided with higher plan and rationale accuracy and less complete reproduction of the misleading recommendation. The confidence results were directionally consistent with closer confidence–accuracy correspondence, but both groups remained overconfident. One possibility is that making an initial judgment made uncertainty more salient before AI exposure and thereby supported metacognitive monitoring. An alternative explanation is more mechanical: higher accuracy in Group A necessarily improves absolute calibration error and Brier scores even without a change in metacognitive awareness. Because the study did not directly measure metacognitive monitoring or uncertainty awareness, these explanations cannot be separated. Process-tracing measures are needed to determine whether participants compared evidence deliberately, anchored on their initial answer, ignored the recommendation, or arrived at the same response through another route.

### 5.3 No statistically detectable sequence difference under expert-keyed recommendations

Judging first could have reduced responsiveness to useful advice by anchoring participants on their initial answer. The expert-keyed-recommendation comparisons addressed this possibility.

No statistically significant sequence difference appeared in expert-keyed joint accuracy or the alignment score under expert-keyed recommendations. The confidence interval for final accuracy remained compatible with modest effects in either direction, so replication with an equivalence-oriented design would be informative.

The independent-first sequence is best understood as a way of ordering human and AI input rather than as a general restriction on AI use. Its value will depend on the cost of delayed advice, the quality distribution of real outputs, and the user’s expertise.

### 5.4 Quality-contingent reliance and task design

The plan–rationale format shows why the sequence matters for pedagogical judgment. A participant could choose an apparently reasonable action while endorsing a rationale that misread the classroom evidence. Under misleading recommendations, judging first was associated with higher expert-keyed accuracy in both components, indicating that the result was not merely an artifact of a stricter composite score.

The findings describe quality-contingent response patterns: expert-keyed recommendations were associated with higher accuracy, whereas the timing advantage emerged when the recommendation was misleading. This pattern is consistent with appropriate reliance as a guiding framework, while the observed indicators remain behavioral outcomes rather than a latent reliance measure.

Under misleading recommendations, the sequence affected both the selected instructional action and the rationale used to justify it. This extends cognitive-forcing research to judgments in which a response and its evidence-based justification must both be appropriate.

### 5.5 Implications for generative AI use in teacher education

The practical relevance is strongest for tasks that require close reading of classroom evidence. Teacher-education programs may consider piloting training sequences that ask preservice teachers to record an initial plan and rationale, review the AI recommendation, identify evidence that supports or contradicts it, and justify any revision. The present study supports evaluation of this workflow; it does not yet establish that the procedure improves authentic classroom decisions or should be adopted as a universal curricular requirement.

AI literacy and competency development can be paired with structured opportunities to evaluate the quality and pedagogical fit of AI output (Ayanwale et al., 2024; Chiu et al., 2024; UNESCO, 2024). Future field trials should test mixed-quality recommendations with practising teachers, authentic planning and diagnostic tasks, live conversational systems, and outcomes that extend beyond multiple-choice scenario judgments.

### 5.6 Limitations and future research

First, the study used a selected set of scenario-based judgments rather than real classroom decisions. The tasks provided experimental control but did not reproduce the time pressure, collaboration, emotional demands, uncertainty, competing information sources, or consequences of authentic teaching. Expert-keyed joint accuracy represented agreement with the panel-designated best response to the supplied evidence, not universally objective pedagogical correctness. Kendall’s W values in the development pool indicated moderate-to-strong rather than uniformly excellent concordance (W = 0.599–0.745), suggesting that some scenario and recommendation judgments remained context-dependent and open to legitimate interpretation. Independent panels and external classroom-performance criteria would strengthen future validation.

Second, the recommendations were generated as drafts with GPT-4 and then standardized through researcher revision and expert review. This design controlled recommendation content but did not reproduce iterative prompting or conversational repair. Perceived AI authorship and credibility were not measured, and the same expert panel reviewed both the answer keys and recommendation texts, creating potential circularity in the content-validation framework. Because the exact original interface prompt and sampling parameters were not archived, regenerating the initial drafts cannot be expected to reproduce the same wording reliably; replicability therefore rests on the archived final standardized stimuli rather than on regeneration of the original model outputs. Moreover, fixed texts developed with a 2025 model do not represent the changing distribution of outputs from all current generative AI systems.

Third, the researcher generated and implemented group allocation in Excel without a separate concealment mechanism, and version assignment was quota-based rather than independently randomized. The manipulation compared two procedures that jointly varied AI-exposure order, self-generation of a pre-AI response, initial commitment, and review structure. Group B spent somewhat longer on the main phase, and Group A did not receive the neutral review prompt used in Groups B and C. Similar estimates in the all-randomized and version-adjusted analyses support robustness within the implemented paradigm but do not isolate which procedural component produced the effect.

Fourth, no formal *a priori* power analysis was conducted, the 12 scenarios formed a fixed item set, and every participant received them in the same order. Item identity and serial position were therefore perfectly confounded. The exploratory quadratic order term suggested modest curvature in accuracy, but response time showed no corresponding linear or quadratic trend; because order and item identity were inseparable, this pattern cannot be attributed specifically to practice or fatigue. Categorical item adjustment and leave-one-item-out analyses support stability within the selected set, but they cannot rule out practice, fatigue, increasing skepticism, or trust recalibration after repeated exposure to mixed-quality recommendations. Broader item-level generalization requires a larger item pool and randomized or counterbalanced order.

Fifth, the no-AI post-test contained only six items and was affected by attrition, so it is best treated as exploratory later performance. Although follow-up completion did not differ significantly by group and completers did not differ significantly from non-completers in age or baseline accuracy, only 26 cleaned-sample participants were non-completers, limiting the precision and sensitivity of the attrition comparisons for small-to-moderate group differences. Prior teaching-related AI use also showed a non-significant imbalance. The sample came from education-related programs at one Chinese university, which limits generalizability across institutions and teacher-education systems.

Finally, matching scores describe observable agreement with the recommendation rather than the reasoning process that produced the answer. The confidence–accuracy results were secondary, both groups showed overconfidence, and lower error in Group A partly reflected higher accuracy. The exploratory training-stage model did not detect moderation, but undergraduate and master’s subgroup estimates were based on smaller samples and therefore had reduced precision; they should not be interpreted as evidence of equivalence across training stages. Prior AI-use covariates did not explain the focal effect, but the study was not powered or preregistered for moderation tests involving AI experience or digital literacy. Future studies should combine randomized component-level manipulations with process tracing, repeated trust and confidence measures, live AI interaction, broader item samples, and preregistered analysis plans.

### 5.7 Conclusion

In this randomized scenario-based experiment, preservice teachers who recorded an independent judgment before viewing an AI recommendation achieved higher expert-keyed joint accuracy when the recommendation was misleading. The difference appeared in both the instructional plan and its rationale, and participants were less likely to reproduce the misleading recommendation. No statistically significant sequence difference was detected under expert-keyed recommendations, but the study was not designed to establish equivalence. Future studies should isolate the procedural components, randomize item order, use live AI interaction, and test more authentic teaching decisions across institutions and educational systems.

## Data Availability

De-identified analysis data may be made available by the corresponding author upon reasonable request.
