# Supplementary material for: Independent judgment before viewing generative AI recommendations reduces susceptibility to misleading advice: a randomized scenario-based experiment with preservice teachers
Source: Front Psychol. 2026 Sep 28;17:1936522. doi: 10.3389/fpsyg.2026.1936522 (PMC13655004; doi:10.3389/fpsyg.2026.1936522)
Supplement: Supplementary file 1 [file Supplementary_file_1.DOCX]

**Supplementary Material 1**

**Experimental materials, development evidence, and additional analyses**

These materials report the experimental conditions, final item set, English translations of the Chinese recommendation texts, the recommendation-generation record, material-development evidence, coding rules, formal item difficulty, model specifications, confidence calibration, post-test effect estimates, and statistical sensitivity analyses. Participants completed the original Chinese materials; the English translations are provided for peer review and reporting purposes.

# S1. Experimental design and judgment–recommendation sequences

Group assignment was randomized 1:1:1. The experimental manipulation compared two implemented judgment–recommendation sequences: an independent response before the recommendation in Group A and the recommendation before the first recorded response in Group B. The procedures also differed in self-generation of a pre-AI answer, initial commitment, and the presence of a neutral review stage. Within each AI group, the first 40 assigned participant numbers received Version X and the next 40 received Version Y to maintain equal quotas. Version allocation was deterministic rather than a second randomization. Recommendation quality was reversed by item across the two versions. The recommendation was displayed once at the designated stage and was not restated in the neutral review prompt or final-response fields. The neutral prompt used in Groups B and C was: “Please reread the scenario and options. You may retain or revise your original judgment.” It supplied no answer key, correctness feedback, or additional recommendation. Because Group A did not receive this neutral prompt, comparisons between Group A and Group C are contextual benchmarks rather than pure AI-versus-no-AI causal contrasts.

| **Group** | **Judgment–recommendation sequence** | **AI recommendation** | **Main items** | **Role in analysis** |
| --- | --- | --- | --- | --- |
| Group A: independent-first | Initial plan/rationale/confidence → recommendation → final judgment | Yes | 12 fixed-order items; 6 correct and 6 incorrect | Core 2 × 2 GEE and benchmark analyses |
| Group B: recommendation-first | Recommendation → first recorded judgment → neutral review → final judgment | Yes | 12 fixed-order items; 6 correct and 6 incorrect | Core 2 × 2 GEE and benchmark analyses |
| Group C: no-AI benchmark | First judgment → neutral review → final judgment | No | 12 fixed-order items | Planned item-adjusted benchmark analyses only |

# Supplementary Figure S1 | Detailed experimental procedure, randomization, and version allocation.

Note. The main manuscript presents a simplified sequence figure. Groups A and B each received six expert-keyed and six misleading recommendations; Group C received no recommendation. Version assignment was quota-based rather than independently randomized.

# S1.1 Descriptive version balance

The table reports background characteristics at assignment. Version was quota-assigned within each AI group; the version-adjusted outcome model is reported in Section S6.2.

| **AI group** | **Version** | **n assigned** | **Baseline joint accuracy** | **Age, M** | **Prior AI for teaching** | **Weekly/daily AI use** |
| --- | --- | --- | --- | --- | --- | --- |
| Group A | X | 40 | 0.554 | 22.58 | 45.0% | 57.5% |
| Group A | Y | 40 | 0.541 | 22.40 | 40.0% | 50.0% |
| Group B | X | 40 | 0.539 | 22.53 | 35.0% | 55.0% |
| Group B | Y | 40 | 0.467 | 22.58 | 25.0% | 60.0% |

# S1.2 Main-phase response time

Timing is descriptive because total time was measured after assignment to the experimental sequence and reflects the full set of screens and responses in each condition.

| **Group** | **n** | **Median total time, min** | **IQR, min** | **Median item time, min** |
| --- | --- | --- | --- | --- |
| Group A | 72 | 33.8 | 28.1–38.2 | 2.8 |
| Group B | 76 | 38.1 | 34.4–43.5 | 3.1 |
| Group C | 70 | 28.3 | 24.1–30.8 | 2.3 |

# S1.3 Participant composition

The randomized cohort comprised students enrolled in teacher-education programs before entry into full-time teaching. Counts for the randomized and cleaned analysis samples are shown below.

| **Characteristic** | **Randomized cohort (n = 240)** | **Cleaned sample (n = 218)** |
| --- | --- | --- |
| Undergraduate, total | 163 | 149 |
| Third-year undergraduate | 71 | 65 |
| Fourth-year undergraduate | 92 | 84 |
| Master’s, total | 77 | 69 |
| First-year master’s | 47 | 43 |
| Second-year master’s | 30 | 26 |
| Primary education | 41 | 39 |
| Teacher education | 39 | 39 |
| Subject teaching: mathematics | 35 | 28 |
| Subject teaching: Chinese | 32 | 28 |
| Subject teaching: English | 26 | 25 |
| Education | 23 | 19 |
| Curriculum and instruction | 22 | 19 |
| Educational technology | 22 | 21 |

# S2. Final scenario set and answer mapping

The formal experiment used 12 main items, six baseline items, and six exploratory post-test items. Correctness required both the selected teaching plan and rationale to match the expert key.

| **ID** | **English item title** | **Pedagogical domain** | **Keyed plan** | **Keyed rationale** | **Version X** | **Version Y** | **Misleading-advice mechanism** |
| --- | --- | --- | --- | --- | --- | --- | --- |
| M02 | Classroom silence and cognitive engagement | Classroom participation, silence, and cognitive engagement | B | D | Expert-keyed | Misleading | Treating silence and brief public responses as evidence of low motivation or low cognitive engagement. |
| M07 | Familiarity and long-term vocabulary retention | Retrieval, spacing, and long-term retention | C | B | Misleading | Expert-keyed | Equating rereading familiarity and immediate recognition with long-term memory. |
| M08 | Should novices solve problems independently immediately? | Worked examples and fading support | D | A | Expert-keyed | Misleading | Withdrawing scaffolding too early from novices who have not yet developed stable procedures. |
| M09 | Can one reading test determine long-term grouping? | Student differences, multiple evidence sources, and dynamic grouping | A | B | Misleading | Expert-keyed | Treating one test result as stable ability and using it to form fixed groups. |
| M10 | Should a high-scoring student be praised as “talented”? | Praise, ability attribution, and process feedback | B | A | Expert-keyed | Misleading | Attributing one high score to stable ability and reinforcing that attribution through identity-based praise. |
| M11 | Do more peer comments indicate effective feedback? | Peer feedback and self-assessment | C | D | Expert-keyed | Misleading | Equating the number of comments and adoptions with feedback quality. |
| M12 | Fast first answers and diagnostic follow-up questions | Teacher questioning, wait time, and diagnostic probing | D | C | Misleading | Expert-keyed | Equating speed, confidence, and majority agreement with conceptual understanding. |
| M13 | A behavior problem or a task-structure problem? | Classroom behavior and task structure | A | C | Expert-keyed | Misleading | Ignoring task structure and attributing situational behavior mainly to stable self-management deficits. |
| M14 | Does online time indicate learning engagement? | Participation in online or blended learning | B | D | Misleading | Expert-keyed | Equating platform-recorded activity with cognitive engagement and learning quality. |
| M16 | Does correct equation balancing show understanding of conservation of mass? | Conceptual understanding and misconception diagnosis | D | B | Expert-keyed | Misleading | Equating procedural accuracy and high analysis accuracy with conceptual understanding. |
| M17 | Does a lively classroom indicate deep cognitive engagement? | Classroom participation and cognitive engagement | A | D | Misleading | Expert-keyed | Equating lively interaction, frequent expression, and interest with deep cognitive engagement. |
| M20 | Does fluency in blocked practice indicate method selection? | Retrieval, spacing, interleaving, and transfer | D | A | Misleading | Expert-keyed | Equating immediate fluency in blocked practice with delayed retention, discrimination, and transfer. |

## S2.1 Baseline and exploratory post-test keys

| **ID** | **English item title** | **Keyed plan** | **Keyed rationale** |
| --- | --- | --- | --- |
| B02 | Reading volume and autonomy support | B | C |
| B03 | Dynamic layers, redundant information, and process understanding | C | B |
| B04 | Copying fluency and delayed retention of poems | D | A |
| B05 | A single English reading score and dynamic grouping | A | C |
| B06 | High-scoring work and ability praise | B | D |
| B07 | Peer and self-assessment in project reports | C | A |
| **ID** | **English item title** | **Keyed plan** | **Keyed rationale** |
| F02 | Worked examples and fading support in experimental design | B | B |
| F03 | Feedback use in revising project reports | C | C |
| F05 | Correlational data and causal explanation | C | A |
| F06 | Does ensemble success indicate mastery by every member? | D | B |
| F07 | Multimedia load in an online museum lesson | A | C |
| F08 | Immediate accuracy and delayed discrimination in map-symbol analysis | B | D |

## S2.2 Original Chinese advice texts and English translations

Participants viewed the Chinese texts shown below. Each item had one expert-keyed recommendation and one recommendation containing the predefined pedagogical error. The English versions are translations for peer review and were not shown to participants.

### M02. Classroom silence and cognitive engagement

**Expert-keyed recommendation — original Chinese:**

沉默本身不足以判断低投入，因为该生的笔记、小组发言和文本回应都显示了认知加工。下一轮可先安排短时书写和同伴准备，再用要求引用证据的追问邀请其表达，同时记录不同参与方式的质量；若突然点名仍受影响，再调整发言支持。

**English translation:**

Silence alone is insufficient evidence of low engagement because the student’s notes, small-group contributions, and written responses all show cognitive processing. In the next lesson, the teacher could first provide brief writing and peer-preparation time, then invite the student to respond through evidence-based follow-up questions while recording the quality of different forms of participation. If sudden public questioning still interferes, the teacher could adjust the support for oral participation.

**Misleading recommendation — original Chinese:**

该生长期少举手且被点名时回答简短，说明公开表达投入不足。下一轮可提高口头参与权重，并安排每节课至少一次随机点名回答，教师及时肯定其发言；持续增加公开表达机会，有助于形成参与习惯，也便于教师直观看到学习状态。

**English translation:**

The student rarely volunteers and gives short answers when called on, indicating insufficient engagement in public expression. In the next lesson, the teacher could give greater weight to oral participation and randomly call on the student at least once in each class, affirming each contribution. Repeated public participation will help form a participation habit and allow the teacher to observe the student’s learning state directly.

*Predefined error mechanism: Treating silence and brief public responses as evidence of low motivation or low cognitive engagement.*

### M07. Familiarity and long-term vocabulary retention

**Expert-keyed recommendation — original Chinese:**

重读后的高识别率与一周后的低自由回忆不一致，而分散小测的延迟保持更好，说明当下熟悉感不足以代表长期掌握。可减少连续重读，改用隔日的低风险提取、及时反馈和累积复习，并依据延迟表现调整间隔。

**English translation:**

High recognition immediately after rereading is inconsistent with low free recall one week later, whereas spaced low-stakes quizzes produced better delayed retention. This suggests that immediate familiarity does not demonstrate long-term mastery. Continuous rereading could be reduced and replaced by low-stakes retrieval on different days, timely feedback, cumulative review, and adjustment of intervals based on delayed performance.

**Misleading recommendation — original Chinese:**

学生重读时识别率高且自我感觉熟悉，说明词形和词义联系已经建立；一周后遗忘主要是接触次数不足。可在同一课时增加多轮重读和抄写，并在每轮后立即做识别测验，达到较高正确率后再进入新词，以强化记忆痕迹。

**English translation:**

Students showed high recognition and familiarity while rereading, indicating that word forms and meanings were already connected; forgetting after one week mainly reflects insufficient exposure. The teacher could add several rounds of rereading and copying within the same lesson, with immediate recognition tests after each round, and introduce new words only after high accuracy is reached.

*Predefined error mechanism: Equating rereading familiarity and immediate recognition with long-term memory.*

### M08. Should novices solve problems independently immediately?

**Expert-keyed recommendation — original Chinese:**

多数新手能解释完整示例，却在独立解题时频繁出现符号错误，而半完成例题提高了成功率，说明其尚未准备好直接撤除支持。可先让学生补全关键步骤并进行自我解释，再依据错误率逐步减少提示，同时为已熟练者提供更快的独立过渡。

**English translation:**

Most novices could explain a complete example but made frequent symbol errors during independent problem solving, while completion problems improved success. They were therefore not ready for the immediate removal of support. Students could first complete key steps and provide self-explanations; prompts could then be reduced according to error rates, with a faster transition for students who were already fluent.

**Misleading recommendation — original Chinese:**

少数学生已经能快速独立完成，说明班级具备从示例转向自主分析的基础。下一轮可取消半完成例题，让所有学生直接解决完整方程，并把错误作为发现薄弱环节的机会；教师在分析结束后统一讲评，以促进独立性和问题解决能力。

**English translation:**

A small number of students could already solve problems quickly and independently, indicating that the class was ready to move from examples to independent work. The teacher could remove completion problems for all students, ask everyone to solve full equations, and use errors to identify weaknesses. A whole-class explanation after the exercise would promote independence and problem-solving ability.

*Predefined error mechanism: Withdrawing scaffolding too early from novices who have not yet developed stable procedures.*

### M09. Can one reading test determine long-term grouping?

**Expert-keyed recommendation — original Chinese:**

一次限时测验低分与口头复述、不同主题文本表现并不一致，不能据此形成长期固定分组。可结合错误类型、口头解释和后续短测建立暂时性教学组，完成针对性教学后重新评估并允许调整；同时保留对解码速度等具体困难的关注。

**English translation:**

A low score on one timed test was inconsistent with the student’s oral retelling and performance on texts with different topics, so it was insufficient for long-term fixed grouping. The teacher could combine error types, oral explanations, and subsequent short assessments to form temporary instructional groups, reassess after targeted teaching, and allow movement between groups while monitoring specific difficulties such as decoding speed.

**Misleading recommendation — original Chinese:**

该生在统一限时测验中得分较低，说明其当前阅读能力与高分学生存在稳定差距。可依据本次成绩进入基础组，集中训练较短文本和答题速度，待下一次单元考试达到标准后再调组；固定一段时间有助于教师实施连续、同层次的教学。

**English translation:**

A low score on the common timed test indicates a stable difference between this student’s current reading ability and that of high-scoring students. The student could be placed in a basic group for shorter texts and speed practice and moved only after meeting the standard on the next unit test. Keeping the group fixed for a period would support continuous same-level instruction.

*Predefined error mechanism: Treating one test result as stable ability and using it to form fixed groups.*

### M10. Should a high-scoring student be praised as “talented”?

**Expert-keyed recommendation — original Chinese:**

首次高分不足以说明稳定天赋，而学生在具体策略反馈后能够有效修改，接受“有天赋”评价后却回避更难任务。教师可肯定作品中的具体效果，并指出构图策略、尝试过程和下一步可调整之处；同时提供有适度挑战的再次选择。

**English translation:**

One high score is insufficient evidence of stable talent. The student revised effectively after receiving specific strategy feedback but avoided harder tasks after being described as talented. The teacher could acknowledge specific strengths in the work, identify the compositional strategy and effort involved, indicate the next adjustable step, and offer another task with a moderate level of challenge.

**Misleading recommendation — original Chinese:**

该生首次作品成绩最高，说明其在构图方面具有明显优势。教师可公开肯定其艺术天赋，并安排其示范成功作品，以增强自信和榜样作用；随后让其继续完成相近难度的构图任务，先稳定优势表现，再逐步增加挑战，避免过早受挫。

**English translation:**

The student achieved the highest score on the first work, showing a clear advantage in composition. The teacher could publicly affirm the student’s artistic talent and ask the student to demonstrate the successful work, thereby strengthening confidence and providing a model for peers. The student could then continue with tasks of similar difficulty before challenges are gradually increased.

*Predefined error mechanism: Attributing one high score to stable ability and reinforcing that attribution through identity-based praise.*

### M11. Do more peer comments indicate effective feedback?

**Expert-keyed recommendation — original Chinese:**

评论数量较多却主要停留在表扬、错别字和句子通顺，修改稿也只出现表层变化；共同分析范文后，反馈开始具体。下一轮可先用标准和范例校准，再要求每条意见引用文本证据、提出可执行修改，并让作者说明采纳或不采纳的理由。

**English translation:**

Although students produced many comments, most concerned praise, typographical errors, and sentence fluency, and revisions remained superficial. Feedback became more specific after the class analyzed an exemplar together. The next cycle could begin with calibration using criteria and examples, require each comment to cite textual evidence and propose an actionable revision, and ask authors to explain why they accepted or rejected each suggestion.

**Misleading recommendation — original Chinese:**

学生已经能为每篇作文提供六七条评论，说明同伴互评参与充分。下一轮可提高每人必须提交的评论数量，并把评论条数计入课堂成绩，同时要求作者至少采纳三条意见；增加反馈覆盖面和采纳次数，有助于形成稳定的互评习惯。

**English translation:**

Students already provide six or seven comments on each composition, indicating strong participation in peer review. The teacher could increase the required number of comments, count comment frequency toward the course grade, and require authors to adopt at least three suggestions. Increasing feedback coverage and the number of adopted comments will help establish stable peer-review habits.

*Predefined error mechanism: Equating the number of comments and adoptions with feedback quality.*

### M12. Fast first answers and diagnostic follow-up questions

**Expert-keyed recommendation — original Chinese:**

首个快速答案使其他学生重复同一解释，而画图追问暴露了对闭合回路的误解；短暂等待和同伴讨论又产生了替代解释。教师可先留出独立思考时间，让所有学生画出路径，再用反例和理由追问比较不同假设，避免只依据最快回答判断全班理解。

**English translation:**

The first quick answer led other students to repeat the same explanation, while a drawing-based follow-up exposed misunderstanding of the closed circuit; brief wait time and peer discussion produced alternative explanations. The teacher could first allow independent thinking, ask all students to draw the current path, and then compare hypotheses through counterexamples and requests for reasons rather than judging the whole class from the fastest response.

**Misleading recommendation — original Chinese:**

首位学生能够迅速提出“电池没电”，说明他已抓住最常见故障，也为同伴提供了有效线索。教师可优先请反应快的学生连续回答，再让其他学生判断赞同与否；若多数人选择相同解释，就直接示范更换电池并进入下一内容，以保持课堂节奏。

**English translation:**

The first student quickly proposed that the battery was dead, showing recognition of the most common fault and providing a useful clue for peers. The teacher could invite quick responders to answer first and ask the class whether they agree. If most students select the same explanation, the teacher could demonstrate battery replacement and move to the next topic to maintain lesson pace.

*Predefined error mechanism: Equating speed, confidence, and majority agreement with conceptual understanding.*

### M13. A behavior problem or a task-structure problem?

**Expert-keyed recommendation — original Chinese:**

偏离任务集中在领材料、换角色和等待工具时，同一学生在结构清楚的阶段能够持续投入，说明行为与任务安排有关。教师可分组预置材料、明确角色与转换信号，并同时说明作品标准，再观察这些调整后行为和学习表现是否改变。

**English translation:**

Off-task behavior occurred mainly while students collected materials, changed roles, or waited for tools, and the same students remained engaged when the task structure was clear. This suggests that the behavior was related to task organization. The teacher could prepare materials by group, specify roles and transition signals, clarify product criteria, and then observe whether behavior and learning change after these adjustments.

**Misleading recommendation — original Chinese:**

几名学生在制作阶段反复交谈、离座和争抢材料，说明其自我管理不足。下一轮可保留原任务流程，为这些学生设置个人行为积分、座位限制和即时提醒，并在活动结束后根据安静程度给予奖励；持续监督有助于建立稳定的课堂规范。

**English translation:**

Several students repeatedly talked, left their seats, and competed for materials during construction, indicating poor self-management. The teacher could retain the existing task sequence, introduce individual behavior points, seating restrictions, and immediate reminders, and provide rewards based on quiet behavior at the end. Continued monitoring would help establish stable classroom norms.

*Predefined error mechanism: Ignoring task structure and attributing situational behavior mainly to stable self-management deficits.*

### M14. Does online time indicate learning engagement?

**Expert-keyed recommendation — original Chinese:**

登录时长和点击次数与发帖质量、小测解释并不一致，网络条件还会造成记录缺失。教师可把平台数据作为初步筛查，结合论证质量、测验、离线学习产物和访问条件判断参与；在提醒低活跃学生前，应先核实其学习方式和实际困难。

**English translation:**

Login duration and click counts were inconsistent with the quality of posts and explanations on quizzes, and network conditions also produced missing records. Platform data could be used as an initial screen but should be combined with argument quality, assessment responses, offline products, and access conditions. Before reminding students with low recorded activity, the teacher should verify their learning methods and practical difficulties.

**Misleading recommendation — original Chinese:**

在线时间和页面点击是平台自动记录的客观指标，比教师主观判断更稳定。教师可按登录时长、点击次数和发帖数量计算参与分，并向低于标准的学生发送提醒，要求增加在线时间；统一阈值便于及时识别投入不足者并维持课程参与。

**English translation:**

Online duration and page clicks are objective platform records and are more stable than teachers’ subjective judgments. The teacher could calculate a participation score from login duration, click counts, and number of posts and require students below the threshold to increase online time. A common threshold would identify insufficient engagement quickly and maintain course participation.

*Predefined error mechanism: Equating platform-recorded activity with cognitive engagement and learning quality.*

### M16. Does correct equation balancing show understanding of conservation of mass?

**Expert-keyed recommendation — original Chinese:**

学生能配平方程式，却认为密闭反应产生的气体会消失，粒子图也未表示气体去向，说明程序性正确不足以确认概念理解。可让学生先预测开放与密闭系统的质量变化，再结合称量证据和粒子图解释物质去向，并根据解释是否修正决定后续支持。

**English translation:**

Students could balance equations but believed that gas produced in a closed reaction disappeared, and their particle diagrams did not show where the gas went. Procedural correctness was therefore insufficient evidence of conceptual understanding. Students could predict mass changes in open and closed systems, use measurement evidence and particle diagrams to explain where matter goes, and receive further support according to whether their explanations change.

**Misleading recommendation — original Chinese:**

学生能够正确配平多数方程式，说明质量守恒的基本规则已经掌握；密闭容器题出错更可能是分析不熟。可增加不同反应类型的配平和质量计算题，要求按固定步骤检查反应物与生成物系数，待正确率稳定后再补充气体实验，以提高解题熟练度。

**English translation:**

Students correctly balance most equations, indicating that the basic rule of conservation of mass has been mastered; errors on closed-container questions are more likely to reflect insufficient practice. The teacher could add equation-balancing and mass-calculation exercises across reaction types, require a fixed checking procedure, and introduce gas experiments only after accuracy becomes stable.

*Predefined error mechanism: Equating procedural accuracy and high analysis accuracy with conceptual understanding.*

### M17. Does a lively classroom indicate deep cognitive engagement?

**Expert-keyed recommendation — original Chinese:**

发言热烈并不等于形成了深入论证，因为多数观点缺少资料依据和对反方证据的回应，而书面比较显示不同学生的加工质量。可先让学生独立整理主张、证据与反例，再进行有规则的回应式讨论，并要求讨论后修正原判断。

**English translation:**

Lively participation did not demonstrate deep argumentation because most contributions lacked documentary evidence and responses to counterarguments, while written comparisons revealed differences in processing quality. Students could first organize claims, evidence, and counterexamples independently, then participate in a structured response-based discussion and revise their initial judgment afterward.

**Misleading recommendation — original Chinese:**

学生发言踊跃且愿意表达个人经验，说明当前辩论已经有效调动了深层参与。下一轮可延长自由辩论时间，并把发言次数、回应速度和观点数量计入参与评价，鼓励学生保持高频互动；较活跃的互动有助于学生接触更多观点并形成更完整的认识。

**English translation:**

Students spoke enthusiastically and shared personal experiences, indicating that the debate had successfully activated deep engagement. The teacher could extend free debate, include frequency, speed of response, and number of ideas in participation assessment, and encourage continued high-frequency interaction. More active discussion will expose students to more perspectives and support fuller understanding.

*Predefined error mechanism: Equating lively interaction, frequent expression, and interest with deep cognitive engagement.*

### M20. Does fluency in blocked practice indicate method selection?

**Expert-keyed recommendation — original Chinese:**

分块分析带来较高的即时速度，却没有稳定支持延迟情境中的方法选择；间隔混合题虽更费力，但辨别逐渐改善。教师可保留新题型初学时的短时集中分析，随后加入分散的混合任务、方法选择说明和延迟检查，以兼顾程序熟练与辨别迁移。

**English translation:**

Blocked practice produced high immediate speed but did not reliably support method selection in delayed contexts; spaced mixed problems were more difficult but gradually improved discrimination. The teacher could use brief blocked practice during initial learning, then introduce distributed mixed tasks, require explanations of method choice, and include delayed checks to support both procedural fluency and transfer.

**Misleading recommendation — original Chinese:**

学生在分块分析中正确率高且速度快，说明各类函数程序正在形成自动化。下一轮应继续按题型安排较大数量的连续分析，并以即时正确率和完成速度作为掌握标准；待每种方法都足够熟练后再统一进行混合测验，可避免过早交错造成混淆和挫败。

**English translation:**

Students were fast and accurate during blocked practice, indicating that procedures for each function type were becoming automatic. The teacher should continue large sets of consecutive problems by type and use immediate accuracy and speed as mastery criteria. Mixed testing could be postponed until each method is fluent, avoiding confusion and frustration from premature interleaving.

*Predefined error mechanism: Equating immediate fluency in blocked practice with delayed retention, discrimination, and transfer.*

# S2.3 Recommendation-generation record

Project records indicate that GPT-4 was used to generate the initial recommendation drafts on December 20, 2025. The exact original interface prompt and platform-specific sampling settings were not archived. The templates below reconstruct the documented generation instructions and are provided for transparency; they should not be read as verbatim transcripts of the original prompt.

**Reconstructed template for a expert-keyed recommendation:** Using the classroom scenario, response options, expert-keyed teaching plan, and expert-keyed rationale provided below, draft one concise and professionally worded pedagogical recommendation. Identify the most defensible instructional action and justify it with the key evidence in the scenario. Do not mention that the response is the “correct” answer.

**Reconstructed template for an inexpert-keyed recommendation:** Using the same classroom scenario and response options, draft one concise, coherent, and professionally worded pedagogical recommendation that incorporates the predefined pedagogical error mechanism. The recommendation should remain superficially plausible and actionable, but its central reasoning should conflict with the expert-keyed plan or rationale. Do not make the error obviously absurd, and do not label the recommendation as incorrect.

Researcher revision and expert review: The researcher aligned expert-keyed drafts with the panel-designated plan and rationale and aligned misleading drafts with one predefined pedagogical error mechanism, while matching length, tone, and professional style as closely as possible. A text was revised when experts judged that the intended error was not clearly codable, surface plausibility or misleading potential was too low, or the paired texts differed noticeably in length, tone, or professionalism. The expert panel then reviewed accuracy, contextual fit, degree of error, surface plausibility, misleading potential, and language comparability. The final standardized texts, rather than unedited model outputs, were used as the experimental stimuli. The original GPT-4 model was not rerun or replaced with a newer model when the final stimuli were standardized.

# S3. Expert review and content-validity evidence

| **ID** | **Expertise** | **AI-education familiarity** |  |
| --- | --- | --- | --- |
| E01 | Educational psychology; learning and cognition | Moderate |  |
| E02 | Teacher education; preservice teacher preparation | Moderate |  |
| E03 | Curriculum and instruction; lesson design | Low–moderate |  |
| E04 | Secondary classroom diagnosis and improvement | Low–moderate |  |
| E05 | Educational measurement and test development | Moderate |  |
| E06 | Educational technology and AI in education | High |  |
| E07 | Primary/lower-secondary teaching and school-based research | Low |  |
| **Rating dimension** | | | **Operational definition** |
| Scenario authenticity | | | 1 = clearly unrealistic; 3 = plausible but requiring revision; 5 = highly consistent with common classrooms. |
| Information sufficiency | | | 1 = insufficient or seriously redundant; 3 = adequate for judgment; 5 = sufficient while retaining reasonable uncertainty. |
| Language clarity | | | 1 = difficult or ambiguous; 3 = minor clarification needed; 5 = natural and clear. |
| Relevance to the research aim | | | 1 = does not assess the target construct; 3 = partly relevant; 5 = highly relevant. |
| Appropriateness of the keyed plan | | | 1 = clearly inappropriate; 3 = debatable; 5 = best fits the key evidence. |
| Accuracy of the keyed rationale | | | 1 = incorrect; 3 = partly sufficient; 5 = accurately integrates the key evidence. |
| Answer-key uniqueness | | | 1 = two equally plausible answers; 3 = boundaries need strengthening; 5 = the best answer is comparatively clear. |
| Expert-keyed-advice accuracy | | | 1 = central judgment is wrong; 3 = broadly correct but needs revision; 5 = accurate. |
| Expert-keyed-advice contextual fit | | | 1 = generic or detached from the scenario; 3 = partly fitted; 5 = uses the key evidence. |
| Degree of error in inexpert-keyed advice | | | 1 = no substantive error; 3 = one identifiable central error; 5 = clear and codable error. |
| Surface plausibility of inexpert-keyed advice | | | 1 = implausible/easy to reject; 3 = moderately attractive; 5 = fluent, professional, and actionable. |
| Language comparability | | | 1 = major differences in length, tone, or professionalism; 3 = broadly matched; 5 = highly matched. |
| Misleading potential | | | 1 = unlikely to be accepted by target participants; 3 = some may accept; 5 = realistically and moderately misleading. |

Experts also provided a separate overall recommendation for each item: delete, major revision, minor revision, retain, or priority retain.

All expert ratings used a 1–5 scale. For CVI calculation, ratings of 4 or 5 were treated as agreement that the dimension was acceptable.

| **Index** | **Value** |
| --- | --- |
| Overall S-CVI/Ave for non-deleted candidate items | 0.887 |
| Main-item S-CVI/Ave | 0.853 |
| Baseline-item S-CVI/Ave | 0.980 |
| Post-test-item S-CVI/Ave | 0.862 |
| Candidate items retained after review | 36 of 40 |
| Items deleted or replaced | 4 |

| **ID** | **I-CVI relevance** | **I-CVI keyed response** | **Composite AI-quality CVI** | **Final disposition** |
| --- | --- | --- | --- | --- |
| M02 | 1 | 1 | 1 | Retained for formal item set |
| M07 | 1 | 1 | 1 | Retained for formal item set |
| M08 | 1 | 1 | 0.95 | Retained for formal item set |
| M09 | 1 | 1 | 1 | Retained for formal item set |
| M10 | 1 | 1 | 0.98 | Retained for formal item set |
| M11 | 0.86 | 0.71 | 0.71 | Retained for formal item set |
| M12 | 1 | 1 | 1 | Retained for formal item set |
| M13 | 1 | 1 | 1 | Retained for formal item set |
| M14 | 1 | 1 | 0.98 | Retained for formal item set |
| M16 | 1 | 1 | 0.98 | Retained for formal item set |
| M17 | 1 | 1 | 1 | Retained for formal item set |
| M20 | 1 | 1 | 0.98 | Retained for formal item set |

For each of the six AI-quality dimensions, ratings of 4 or 5 were coded as agreement. The composite AI-quality CVI is the proportion of acceptable ratings across the six dimensions and seven experts for each item.

Across the 12 retained main items, relevance I-CVI ranged from 0.86 to 1.00, keyed-response I-CVI ranged from 0.71 to 1.00, and composite AI-quality CVI ranged from 0.71 to 1.00. M11 was the only retained item below 0.78 for the keyed response and composite AI-quality index; its leave-one-item-out analysis is reported in Section S6.2.

The retained keys represent the expert panel’s most defensible response to the evidence presented in each scenario, not uniquely correct solutions across all classroom contexts.

## S3.1 Inter-rater concordance beyond CVI

Kendall’s coefficient of concordance (W) was calculated as an ordinal agreement index across the full candidate-item development pool. The scenario, answer-key, and overall-retainability dimensions used all 40 candidate items; the six AI-recommendation dimensions used the 20 main candidate items for which AI texts were rated. W evaluates concordance in the experts’ relative ordering of items and complements, rather than replaces, CVI threshold agreement. Values ranged from 0.599 to 0.745, and all tests were significant at p < .001. These values indicate moderate-to-strong rather than uniformly excellent concordance and are consistent with some context dependence in the underlying pedagogical judgments.

| **Rating dimension** | **Candidate items** | **Kendall’s W** | **χ²** | **df** | **p** |
| --- | --- | --- | --- | --- | --- |
| Scenario authenticity | 40 | 0.647 | 176.75 | 39 | < .001 |
| Information sufficiency | 40 | 0.618 | 168.72 | 39 | < .001 |
| Language clarity | 40 | 0.599 | 163.54 | 39 | < .001 |
| Relevance to research aim | 40 | 0.628 | 171.46 | 39 | < .001 |
| Appropriateness of keyed plan | 40 | 0.718 | 195.97 | 39 | < .001 |
| Accuracy of keyed rationale | 40 | 0.645 | 176.01 | 39 | < .001 |
| Answer-key uniqueness | 40 | 0.726 | 198.32 | 39 | < .001 |
| Expert-keyed advice accuracy | 20 | 0.624 | 82.93 | 19 | < .001 |
| Expert-keyed advice contextual fit | 20 | 0.657 | 87.41 | 19 | < .001 |
| Misleading-advice error degree | 20 | 0.642 | 85.38 | 19 | < .001 |
| Misleading-advice surface plausibility | 20 | 0.664 | 88.37 | 19 | < .001 |
| Advice language comparability | 20 | 0.738 | 98.12 | 19 | < .001 |
| Misleading potential | 20 | 0.658 | 87.46 | 19 | < .001 |
| Overall retainability | 40 | 0.745 | 203.41 | 39 | < .001 |

Because the final 12 main items were selected after revision and screening, their ratings had a restricted range; the concordance analysis therefore uses the full development pool. The same panel evaluated the answer keys and recommendation texts, so these statistics quantify within-panel consistency rather than independent external validation.

# S4. Cognitive interviews and pilot screening

Eight teacher-education students completed cognitive interviews. The table reports response-process and pilot evidence for the 12 items retained in the formal main experiment.

| **ID** | **Understanding rate** | **Key evidence noticed** | **Answer ambiguity rate** | **Mean realism** | **Mean reading burden** | **AI-language difference rate** |
| --- | --- | --- | --- | --- | --- | --- |
| M02 | 1 | 0.875 | 0.25 | 5 | 2.25 | 0.125 |
| M07 | 1 | 0.625 | 0.125 | 4.75 | 2 | 0.25 |
| M08 | 0.875 | 0.875 | 0.125 | 4.88 | 2.12 | 0.125 |
| M09 | 0.75 | 0.875 | 0.25 | 4.5 | 2.25 | 0.375 |
| M10 | 0.875 | 0.5 | 0.125 | 5 | 2.12 | 0.25 |
| M11 | 0.875 | 0.875 | 0.125 | 4.75 | 2.25 | 0.375 |
| M12 | 0.75 | 0.75 | 0.125 | 4.88 | 2.12 | 0.375 |
| M13 | 1 | 0.625 | 0 | 4.88 | 2 | 0 |
| M14 | 1 | 0.625 | 0.25 | 5 | 2.12 | 0 |
| M16 | 0.625 | 0.625 | 0.25 | 4.88 | 2.62 | 0.125 |
| M17 | 0.875 | 0.75 | 0.25 | 4.88 | 2.25 | 0.125 |
| M20 | 1 | 0.625 | 0.375 | 4.88 | 2.12 | 0.125 |

Rates are proportions across eight interviews. Understanding rate was the proportion of interviewees who understood the scenario; key evidence noticed was the proportion who identified at least one key piece of evidence; answer ambiguity rate was the proportion who regarded two response options as similarly defensible; and AI-language difference rate was the proportion who detected a difference in language quality between the correct and inexpert-keyed recommendations. Realism and reading burden were rated on five-point scales, from 1 = very low to 5 = very high.

| **Pilot characteristic** | | | | **Distribution** | | | | |
| --- | --- | --- | --- | --- | --- | --- | --- | --- |
| Records | | | | 48 | | | | |
| Completion | | | | 44 complete; 1 invalid; 3 partially complete | | | | |
| Attention check | | | | 45 passed; 3 failed | | | | |
| Gender | | | | 36 women; 9 men; 3 other/not reported | | | | |
| Year of study | | | | 17 fourth-year undergraduate; 14 third-year undergraduate; 14 first-year master’s; 3 second-year master’s | | | | |
| Teaching-practicum experience | | | | 25 limited; 13 moderate; 10 none | | | | |
| AI-use frequency | | | | 18 monthly; 23 weekly; 3 daily; 4 rarely | | | | |
| **ID** | **Valid n** | **Joint accuracy** | **Misleading-advice matching** | **Expert-keyed-advice alignment** | **Credibility gap** | **Context realism** | **Difficulty** | **Final disposition** |
| M02 | 48 | 0.667 | 0.25 | 0.875 | 0.875 | 4.083 | 2.729 | Retained for formal study |
| M07 | 45 | 0.489 | 0.318 | 0.739 | 0.415 | 4.156 | 3 | Retained for formal study |
| M08 | 48 | 0.604 | 0.208 | 0.875 | 0.5 | 4.021 | 2.958 | Retained for formal study |
| M09 | 46 | 0.63 | 0.318 | 0.792 | 0.86 | 4.043 | 2.978 | Retained for formal study |
| M10 | 48 | 0.521 | 0.333 | 0.875 | 0.75 | 4.104 | 3.25 | Retained for formal study |
| M11 | 47 | 0.638 | 0.042 | 0.957 | 2.19 | 3.979 | 3.106 | Retained for peer-feedback coverage; leave-one-item-out sensitivity reported in S6.2 |
| M12 | 48 | 0.604 | 0.292 | 0.875 | 0.875 | 3.917 | 3.042 | Retained for formal study |
| M13 | 47 | 0.638 | 0.261 | 0.708 | 0.824 | 3.915 | 2.872 | Retained for formal study |
| M14 | 48 | 0.562 | 0.375 | 0.792 | 0.75 | 4.021 | 3.271 | Retained for formal study |
| M16 | 47 | 0.745 | 0.217 | 0.667 | 0.433 | 3.596 | 3.298 | Retained for formal study |
| M17 | 47 | 0.617 | 0.417 | 0.739 | 0.837 | 4.064 | 3.213 | Retained for formal study |
| M20 | 48 | 0.479 | 0.458 | 0.833 | 0.292 | 4.083 | 3.042 | Retained for formal study |

Credibility gap was calculated as the mean credibility rating for expert-keyed recommendations minus the mean credibility rating for inexpert-keyed recommendations; positive values indicate higher perceived credibility for the expert-keyed recommendation. Pilot values are descriptive screening evidence. M11 showed the weakest balance (inexpert-keyed-advice matching = 0.042; credibility gap = 2.190) and was retained to preserve coverage of peer-feedback judgment. Excluding M11 produced OR = 1.975, 95% CI [1.447, 2.695], p < .001 for the focal inexpert-keyed-advice comparison.

# S5. Coding rules and formal experiment results

| **Measure** | **Coding** | **Interpretation** |
| --- | --- | --- |
| Expert-keyed joint accuracy | 1 only if both final plan and final rationale matched the expert key; otherwise 0 | Primary binary outcome |
| Expert-keyed-advice alignment | Final response matches neither/one/both advice components, normalized to 0/0.5/1 | Behavioral alignment; not causal uptake in Group B |
| Misleading-advice non-alignment | 1 − normalized matching score | Non-alignment with the misleading recommendation; may be correct or another error |
| Complete matching | Both final components match the recommendation | Binary alignment outcome |
| Harmful change (Group A) | Initial joint correct → final joint incorrect under inexpert-keyed advice | Loss of a correct pre-AI answer |
| Beneficial change (Group A) | Initial joint incorrect → final joint correct under expert-keyed advice | Correction after expert-keyed advice |
| Absolute confidence–accuracy error | \|confidence/100 − final joint correctness\| | Magnitude of confidence–correctness discrepancy |
| Final plan accuracy | 1 if the final plan matched the expert key; otherwise 0 | Secondary component outcome |
| Final rationale accuracy | 1 if the final rationale matched the expert key; otherwise 0 | Secondary component outcome |

## S5.1 Sample flow and baseline checks

| **Stage** | | **Group A** | **Group B** | **Group C** | **Total** |
| --- | --- | --- | --- | --- | --- |
| Randomized | | 80 | 80 | 80 | 240 |
| Attention-check failure | | 4 | 2 | 6 | 12 |
| Extremely short response pattern | | 4 | 2 | 4 | 10 |
| Included in main analysis | | 72 | 76 | 70 | 218 |
| Included in post-test analysis | | 68 | 64 | 60 | 192 |
| **Variable** | **Group A** | **Group B** | **Group C** | **Test** | **p** |
| Age, M (SD) | 22.611 (1.812) | 22.553 (1.792) | 22.443 (1.708) | F = 0.165 | .848 |
| Baseline joint accuracy, M (SD) | 0.539 (0.234) | 0.505 (0.216) | 0.528 (0.213) | F = 0.444 | .642 |
| Gender: female, n (%) | 50 (69.4%) | 48 (63.2%) | 47 (67.1%) | χ² = 2.928 | .570 |
| Gender: male, n (%) | 20 (27.8%) | 27 (35.5%) | 23 (32.9%) |  |  |
| Gender: other/not reported, n (%) | 2 (2.8%) | 1 (1.3%) | 0 (0.0%) |  |  |
| AI-use frequency: rarely, n (%) | 11 (15.3%) | 12 (15.8%) | 10 (14.3%) | χ² = 2.810 | .832 |
| AI-use frequency: monthly, n (%) | 23 (31.9%) | 21 (27.6%) | 21 (30.0%) |  |  |
| AI-use frequency: weekly, n (%) | 28 (38.9%) | 26 (34.2%) | 29 (41.4%) |  |  |
| AI-use frequency: daily, n (%) | 10 (13.9%) | 17 (22.4%) | 10 (14.3%) |  |  |
| Teaching practicum: none, n (%) | 18 (25.0%) | 21 (27.6%) | 13 (18.6%) | χ² = 4.973 | .290 |
| Teaching practicum: limited, n (%) | 35 (48.6%) | 44 (57.9%) | 39 (55.7%) |  |  |
| Teaching practicum: moderate, n (%) | 19 (26.4%) | 11 (14.5%) | 18 (25.7%) |  |  |
| Prior AI use for teaching-related tasks: no, n (%) | 41 (56.9%) | 53 (69.7%) | 34 (48.6%) | χ² = 6.873 | .032 |
| Prior AI use for teaching-related tasks: yes, n (%) | 31 (43.1%) | 23 (30.3%) | 36 (51.4%) |  |  |

For the cleaned A/B comparison, standardized mean differences were 0.032 for age, 0.149 for baseline joint accuracy, and 0.268 for prior teaching-related AI use. Extreme speeding was defined as total main-phase time below 8 minutes. Exactly 10 participants met this threshold; all were excluded, and no participant at or above 8 minutes was excluded for speeding. Twelve participants failed the instructional attention check. The all-randomized A/B analysis in Section S6.2 retained the speed-flagged cases with available outcomes and therefore evaluates sensitivity to the speeding exclusion.

## S5.2 Formal-study item difficulty and ceiling/floor checks

The table reports expert-keyed joint accuracy for the cleaned main-analysis sample. Because recommendation quality was reversed across versions, item-level values are shown separately for each AI group and recommendation-quality condition. Overall item accuracy ranged from 0.454 to 0.616; no item exceeded 0.90 or fell below 0.10.

| **Item** | **Valid n** | **Overall** | **A correct** | **A incorrect** | **B correct** | **B incorrect** | **C no AI** |
| --- | --- | --- | --- | --- | --- | --- | --- |
| M02 | 213 | 0.601 | 0.727 | 0.583 | 0.737 | 0.405 | 0.580 |
| M07 | 215 | 0.526 | 0.649 | 0.514 | 0.649 | 0.351 | 0.493 |
| M08 | 215 | 0.484 | 0.571 | 0.514 | 0.595 | 0.351 | 0.435 |
| M09 | 218 | 0.546 | 0.649 | 0.543 | 0.684 | 0.368 | 0.514 |
| M10 | 217 | 0.507 | 0.629 | 0.528 | 0.605 | 0.342 | 0.471 |
| M11 | 216 | 0.458 | 0.559 | 0.444 | 0.579 | 0.342 | 0.414 |
| M12 | 213 | 0.568 | 0.703 | 0.545 | 0.658 | 0.389 | 0.551 |
| M13 | 216 | 0.588 | 0.686 | 0.595 | 0.711 | 0.395 | 0.574 |
| M14 | 214 | 0.565 | 0.686 | 0.571 | 0.703 | 0.368 | 0.536 |
| M16 | 218 | 0.454 | 0.543 | 0.459 | 0.579 | 0.368 | 0.386 |
| M17 | 213 | 0.549 | 0.667 | 0.559 | 0.676 | 0.368 | 0.515 |
| M20 | 216 | 0.616 | 0.750 | 0.571 | 0.763 | 0.421 | 0.594 |

M16 had the lowest overall expert-keyed joint accuracy (0.454), and M20 had the highest (0.616). The absence of extreme item means reduces concern about global ceiling or floor effects, although item identity remained confounded with serial position.

## S5.3 Core GEE model and behavioral outcomes

Plan- and rationale-accuracy estimates reported below are from the version-adjusted participant-clustered logistic GEE models used in the main manuscript; other estimates retain their stated model specifications.

| **Comparison** | **Log odds** | **Robust SE** | | **OR** | | **95% CI** | **p** |
| --- | --- | --- | --- | --- | --- | --- | --- |
| Group A vs. Group B under inexpert-keyed advice | 0.648 | 0.155 | | 1.912 | | [1.413, 2.589] | < .001 |
| Correct vs. inexpert-keyed advice within Group B | 1.424 | 0.172 | | 4.154 | | [2.965, 5.820] | < .001 |
| Group A × expert-keyed-advice interaction | −0.845 | 0.224 | | 0.429 | | [0.277, 0.666] | < .001 |
| Group A vs. Group B under expert-keyed advice | −0.197 | 0.162 | | 0.821 | | [0.598, 1.128] | .225 |
| Baseline joint accuracy (per 0.10 increase) | 0.429 | 0.034 | | 1.536 | | [1.438, 1.641] | < .001 |
| Item order | 0.010 | 0.015 | | 1.010 | | [0.981, 1.040] | .500 |
| Group A vs. Group B under inexpert-keyed advice, version adjusted | 0.643 | 0.155 | | 1.901 | | [1.403, 2.577] | < .001 |
| Group A vs. Group B under expert-keyed advice, version adjusted | −0.204 | 0.161 | | 0.815 | | [0.595, 1.117] | .204 |
| **Condition/outcome** | **Group A** | | **Group B** | | **Estimate** | | **p** |
| Expert-keyed-advice alignment score | 0.767 | | 0.781 | | Adjusted difference = −0.038; SE = 0.022 | | .086 |
| Misleading-advice non-alignment score | 0.702 | | 0.510 | | Adjusted difference = 0.172; SE = 0.033 | | < .001 |
| Complete matching with inexpert-keyed advice | 0.258 | | 0.468 | | OR = 0.403; 95% CI [0.296, 0.549] | | < .001 |
| Absolute confidence–accuracy error under inexpert-keyed advice | 0.419 | | 0.493 | | Adjusted difference = −0.064; SE = 0.015 | | < .001 |
| Harmful change (Group A only) | 0.103 | | N/A | | Descriptive | | — |
| Beneficial change (Group A only) | 0.195 | | N/A | | Descriptive | | — |
| Final plan accuracy, inexpert-keyed recommendation | 0.608 | | 0.439 | | OR = 1.938; 95% CI [1.453, 2.586] | | < .001 |
| Final rationale accuracy, inexpert-keyed recommendation | 0.585 | | 0.406 | | OR = 2.028; 95% CI [1.482, 2.775] | | < .001 |
| Final plan accuracy, expert-keyed recommendation | 0.793 | | 0.788 | | OR = 0.941; 95% CI [0.677, 1.308] | | .717 |
| Final rationale accuracy, expert-keyed recommendation | 0.741 | | 0.774 | | OR = 0.728; 95% CI [0.522, 1.016] | | .062 |
| Other/partially aligned incorrect response | 0.207 | | 0.160 | | Descriptive decomposition | | — |
| Signed confidence bias, inexpert-keyed recommendation | 0.170 | | 0.320 | | Mean confidence/100 − joint accuracy | | — |
| Mean confidence when jointly correct, inexpert-keyed recommendation | 0.767 | | 0.767 | | Descriptive | | — |
| Mean confidence when jointly incorrect, inexpert-keyed recommendation | 0.633 | | 0.648 | | Descriptive | | — |

Under inexpert-keyed recommendations, the mutually exclusive outcome distribution was: expert-keyed joint response (Group A = 0.535; Group B = 0.373), complete reproduction of the misleading recommendation (0.258; 0.468), and another or partially aligned incorrect response (0.207; 0.160). Complete reproduction was the primary behavioral outcome for H2a; absolute confidence–accuracy error was examined under RQ2.

## S5.4 Secondary confidence–accuracy analysis

Under expert-keyed recommendations, absolute confidence–accuracy error was 0.339 in Group A and 0.332 in Group B. Under misleading recommendations, the corresponding values were 0.419 and 0.493; the adjusted difference was −0.064, SE = 0.015, p < .001. Mean confidence was 0.767 in both groups when responses were jointly correct and 0.633 versus 0.648 when responses were jointly incorrect. Signed confidence bias, used as calibration-in-the-large, was 0.170, participant-clustered bootstrap 95% CI [0.120, 0.218], in Group A and 0.320, 95% CI [0.265, 0.375], in Group B. Brier scores were 0.230, 95% CI [0.209, 0.251], and 0.299, 95% CI [0.271, 0.327], respectively. Both groups were overconfident under misleading recommendations. The absolute-error and Brier differences co-occurred with the accuracy difference and should be interpreted as secondary confidence–accuracy correspondence, not definitive evidence of superior metacognitive monitoring.

| **Advice** | **Group** | **Item n** | **Accuracy** | **Mean confidence** | **Signed bias (95% CI)** | **Brier (95% CI)** | **Absolute error (95% CI)** |
| --- | --- | --- | --- | --- | --- | --- | --- |
| Misleading | A | 426 | 0.535 | 0.705 | 0.170 [0.120, 0.218] | 0.230 [0.209, 0.251] | 0.419 [0.395, 0.442] |
| Misleading | B | 451 | 0.373 | 0.692 | 0.320 [0.265, 0.375] | 0.299 [0.271, 0.327] | 0.493 [0.464, 0.523] |
| Expert-keyed | A | 425 | 0.652 | 0.739 | 0.087 [0.035, 0.140] | 0.167 [0.144, 0.192] | 0.339 [0.310, 0.368] |
| Expert-keyed | B | 452 | 0.662 | 0.732 | 0.071 [0.022, 0.122] | 0.161 [0.138, 0.185] | 0.332 [0.306, 0.360] |

**Binned calibration under inexpert-keyed recommendations**

| **Confidence range** | **A n** | **A mean confidence** | **A accuracy** | **B n** | **B mean confidence** | **B accuracy** |
| --- | --- | --- | --- | --- | --- | --- |
| 20-39 | 8 | 0.321 | 0.000 | 10 | 0.340 | 0.000 |
| 40-49 | 28 | 0.451 | 0.036 | 27 | 0.452 | 0.000 |
| 50-59 | 53 | 0.550 | 0.226 | 65 | 0.558 | 0.108 |
| 60-69 | 94 | 0.647 | 0.426 | 117 | 0.644 | 0.282 |
| 70-79 | 124 | 0.748 | 0.653 | 138 | 0.744 | 0.464 |
| 80-89 | 95 | 0.839 | 0.811 | 64 | 0.840 | 0.688 |
| 90-100 | 24 | 0.944 | 0.708 | 30 | 0.947 | 0.667 |

Note. Confidence was divided into the observed ranges shown above. Sparse low-confidence bins should be interpreted descriptively. Positive signed bias indicates overconfidence.

## Supplementary Figure S2 | Binned confidence calibration under misleading AI recommendations.

Note. Points show observed expert-keyed joint accuracy within confidence bins; the dashed diagonal represents perfect calibration. The lowest-confidence bins contained relatively few observations (Group A n = 8 and Group B n = 10 for confidence 20–39), so the curves are descriptive. Cluster-bootstrap interval estimates for aggregate signed bias and Brier scores are reported above.

## S5.5 Planned no-AI benchmark comparisons

| **Comparison** | **Observed means** | **OR** | **95% CI** | **p** |
| --- | --- | --- | --- | --- |
| Group A inexpert-keyed advice vs. Group C | 0.535 vs. 0.505 | 1.125 | [0.864, 1.466] | .380 |
| Group B inexpert-keyed advice vs. Group C | 0.373 vs. 0.505 | 0.577 | [0.436, 0.764] | < .001 |
| Group A expert-keyed advice vs. Group C | 0.652 vs. 0.505 | 2.012 | [1.548, 2.616] | < .001 |
| Group B expert-keyed advice vs. Group C | 0.662 vs. 0.505 | 2.479 | [1.865, 3.296] | < .001 |

# S6. Exploratory post-test and sensitivity analyses

All randomized participants were invited to complete the later no-AI task; completion was voluntary. Complete-case participant-level means and item-adjusted GEE contrasts are reported below before the extreme missing-data sensitivity scenarios.

| **Group** | **n** | **Mean joint accuracy** | **SD** |
| --- | --- | --- | --- |
| A | 68 | 0.472 | 0.241 |
| B | 64 | 0.408 | 0.227 |
| C | 60 | 0.452 | 0.249 |

**Participant-level pairwise effects**

| **Comparison** | **Mean difference** | **95% CI** | **Hedges g** | **p** |
| --- | --- | --- | --- | --- |
| A − B | 0.063 | [-0.017, 0.144] | 0.268 | 0.123 |
| A − C | 0.020 | [-0.066, 0.106] | 0.081 | 0.648 |
| B − C | -0.043 | [-0.128, 0.042] | -0.181 | 0.314 |

**Item-adjusted participant-clustered logistic GEE**

| **Comparison** | **OR** | **95% CI** | **p** |
| --- | --- | --- | --- |
| A vs. B | 1.251 | [0.915, 1.710] | 0.160 |
| A vs. C | 1.050 | [0.754, 1.462] | 0.773 |
| B vs. C | 0.839 | [0.606, 1.163] | 0.293 |

**Extreme attrition assumptions**

| **Analysis** | **Group A** | **Group B** | **Group C** | **Interpretation** |
| --- | --- | --- | --- | --- |
| Cleaned complete-case joint accuracy | 0.472 | 0.408 | 0.452 | Descriptive ordering A > C > B |
| Observed rows from flagged cases included | 0.472 | 0.410 | 0.451 | Similar descriptive ordering |
| Pessimistic assumption: missing coded incorrect | 0.440 | 0.340 | 0.386 | A remained descriptively higher |
| Optimistic assumption: missing coded correct | 0.507 | 0.507 | 0.533 | Ordering changed |

The post-test contained six items. In the complete-case analysis, Group A was directionally above Group B, but the A–B confidence interval included zero and the item-adjusted GEE confidence interval included 1. Under the pessimistic assumption, every missing post-test response was coded incorrect; under the optimistic assumption, every missing response was coded correct. Because the group ordering changed under the optimistic assumption, the result is reported as exploratory later unaided performance rather than stable transfer.

## S6.1 Follow-up completion diagnostics

Follow-up completion was examined within the cleaned main-analysis sample (192 completers and 26 non-completers). Group differences in completion and comparisons on available baseline characteristics were not statistically significant. The comparison involving prior AI use for teaching-related tasks showed a non-significant tendency. Because only 26 cleaned participants did not complete the follow-up, these comparisons had limited precision and should not be treated as evidence that small-to-moderate group differences in completion were absent.

| **Comparison** | **Descriptive result** | **Test** | **p** |
| --- | --- | --- | --- |
| Group distribution among cleaned participants | A: 68/72; B: 64/76; C: 60/70 | χ²(2) = 4.233 | .120 |
| Age, years | Completers: 22.516 (1.740); non-completers: 22.692 (1.975) | Welch t = −0.434 | .667 |
| Baseline joint accuracy | Completers: 0.528 (0.222); non-completers: 0.487 (0.210) | Welch t = 0.933 | .358 |
| Prior AI use for teaching-related tasks | Completers: 84/192 (43.8%); non-completers: 6/26 (23.1%) | χ²(1) = 3.229 | .072 |
| AI-use frequency | Four-category distribution | χ²(3) = 1.161 | .762 |
| Teaching-practicum experience | Three-category distribution | χ²(2) = 1.677 | .432 |

Note. Completion diagnostics use participants who passed the main-analysis cleaning criteria. In the full randomized cohort, follow-up non-completion was 6/80, 13/80, and 11/80 in Groups A, B, and C, respectively, χ²(2) = 2.971, p = .226.

## S6.2 Robustness checks for the focal inexpert-keyed-advice comparison

| **Model/check** | **OR** | **95% CI** | **p** | **Specification** |
| --- | --- | --- | --- | --- |
| Main GEE | 1.912 | [1.413, 2.589] | < .001 | Baseline joint accuracy and linear item order |
| Categorical item fixed effects | 1.935 | [1.422, 2.633] | < .001 | Controls differences among the 12 selected items |
| Prior teaching-related AI use adjusted | 1.905 | [1.404, 2.585] | < .001 | Adds prior teaching-related AI use |
| AI-use frequency adjusted | 1.896 | [1.403, 2.562] | < .001 | Adds general AI-use frequency |
| Both AI-experience variables adjusted | 1.885 | [1.391, 2.554] | < .001 | Adds both AI-use covariates |
| Independent working correlation | 1.916 | [1.415, 2.593] | < .001 | Alternative working correlation |
| Version adjusted | 1.901 | [1.403, 2.577] | < .001 | Adds Version X/Y indicator |
| All randomized A/B available cases | 1.814 | [1.345, 2.448] | < .001 | Retains all 160 randomized A/B participants with observed item outcomes |
| M11 excluded | 1.975 | [1.447, 2.695] | < .001 | Addresses weaker pilot balance for M11 |
| Version X only | 2.152 | [1.383, 3.350] | < .001 | Focal incorrect-recommendation contrast within Version X |
| Version Y only | 1.704 | [1.124, 2.582] | .012 | Focal incorrect-recommendation contrast within Version Y |
| Leave-one-item-out analyses (12 models; version adjusted) | 1.857–1.984 | All lower bounds > 1.32 | All < .001 | Minimum when M14 was excluded; maximum when M16 was excluded |

## S6.3 Exploratory sequence trends and training-stage moderation

The fixed 12-item order was examined descriptively and with exploratory trend models. The table below shows observed expert-keyed joint accuracy and median total response time for Groups A and B by serial position. Because each serial position corresponded to one specific item, these values cannot separate item difficulty from sequence effects.

| Item order | Item ID | Observed expert-keyed joint accuracy | Median total response time, s |
| --- | --- | --- | --- |
| 1 | M02 | 0.611 | 176.5 |
| 2 | M07 | 0.541 | 179.0 |
| 3 | M08 | 0.507 | 177.5 |
| 4 | M09 | 0.561 | 176.5 |
| 5 | M10 | 0.524 | 171.0 |
| 6 | M11 | 0.479 | 182.5 |
| 7 | M12 | 0.576 | 173.5 |
| 8 | M13 | 0.595 | 165.0 |
| 9 | M14 | 0.579 | 181.0 |
| 10 | M16 | 0.486 | 180.0 |
| 11 | M17 | 0.566 | 175.0 |
| 12 | M20 | 0.626 | 177.0 |

In an exploratory participant-clustered logistic GEE that added centered linear and quadratic item-order terms to the core model, the linear term was not significant (B = 0.010, SE = 0.015, p = .511), whereas the quadratic term was small but statistically significant (B = 0.010, SE = 0.005, p = .044). The focal misleading-advice sequence contrast remained essentially unchanged, OR = 1.918, 95% CI [1.415, 2.599], p < .001. A parallel Gaussian GEE for log total response time showed neither a linear order trend (B = −0.001, SE = 0.002, p = .699) nor a quadratic trend (B < 0.001, SE = 0.001, p = .833). Given the perfect confounding of item identity and serial position, the accuracy curvature is not interpreted as evidence of practice or fatigue.

### Exploratory moderation by training stage

Training stage was coded as undergraduate versus master’s level. The cleaned A/B sample contained 48 undergraduate and 24 master’s students in Group A and 55 undergraduate and 21 master’s students in Group B. A participant-clustered logistic GEE added training stage and all two- and three-way interactions with sequence and recommendation quality to the primary model. The sequence × recommendation quality × training-stage interaction was not significant (p = .618).

| Training stage | Group A n | Group B n | A vs. B under misleading advice, OR (95% CI) | p |
| --- | --- | --- | --- | --- |
| Undergraduate | 48 | 55 | 1.832 [1.272, 2.637] | .001 |
| Master’s | 24 | 21 | 2.031 [1.211, 3.408] | .007 |

Both training-stage strata therefore showed the same directional pattern under misleading advice, but the subgroup estimates were less precise than the full-sample estimate. The non-significant three-way interaction should not be interpreted as evidence of equivalence across training stages.
